# A Predictive Computational Framework for *Staphylococcus aureus* Biofilm Growth Stages in Hydrodynamic Conditions

**DOI:** 10.3390/pathogens15010118

**Published:** 2026-01-21

**Authors:** Sarees Shaikh, Abiye Mekonnen, Abdul Nafay Saleem, Patrick Ymele-Leki

**Affiliations:** 1 Department of Chemical Engineering, Howard University, Washington, DC 20059, USA; sarees.shaikh@bison.howard.edu (S.S.);; 2 Department of Electrical Engineering and Computer Science, Howard University, Washington, DC 20059, USA

**Keywords:** *Staphylococcus aureus*, bacteria, biofilm, growth cycle, exodus, shear rate, kinetic modeling

## Abstract

Biofilms formed by *Staphylococcus aureus* on medical devices and tissue surfaces are a major contributor to persistent infections due to their resistance to antibiotics. Hydrodynamic forces in physiological and device-associated environments significantly influence biofilm development, yet the dynamics of detachment and regrowth under flow remain poorly quantified. In this study, biofilm surface coverage was measured in microfluidic flow assays across combinations of shear rates and nutrient concentrations. A computational workflow was used to segment biofilm trajectories into three kinetic phases—growth, exodus, and regrowth—based on surface coverage dynamics. Each phase was modeled using parametric functions, and fitted parameters were interpolated across experimental conditions to reconstruct biofilm lifecycles throughout the flow–nutrient conditions. The analysis revealed that intermediate shear rates triggered early detachment events while suppressing subsequent regrowth, whereas lower and higher shear regimes favored biofilm persistence. The resulting model enables quantitative comparison of condition-specific biofilm behaviors and identifies key thresholds in mechanical and nutritional inputs that modulate biofilm stability. These findings establish a phase-resolved framework for studying *S. aureus* biofilms under hydrodynamic stress and support future development of targeted strategies to control biofilm progression in clinical and engineered systems.

## 1. Introduction

*Staphylococcus aureus* is a major human pathogen capable of forming persistent biofilms on host tissues and implanted medical devices. Within these communities, bacterial cells embed themselves in a protective extracellular matrix that enhances resistance to antimicrobials and host immune clearance [1,2,3]. This biofilm-associated protection enables the bacteria to persist in the host and often leads to chronic infections or relapsing, device-related infections [1]. This can allow *S. aureus* to evade phagocytosis and resist antimicrobial treatment, establishing reservoirs that can seed continuous or secondary infections [4]. As a result, understanding the physical and environmental determinants of *S. aureus* biofilm development remains a critical challenge in biomedical engineering and infectious disease research.

Fluid shear stress is a key factor governing biofilm architecture, stability, and detachment. Previous works have shown that shear modulates both the initial adhesion of *S. aureus* to surfaces (such as collagen) [1,5] and the detachment of surface-bound cells, particularly when shear interacts with host-targeting monoclonal antibodies [5]. Biofilms grown under physiologically relevant shear can undergo erosion and active shedding, releasing bacterial cells with altered adhesive properties [6]. Nutrient availability is another factor that influences *S. aureus* biofilm dynamics through metabolic cues and quorum-sensing regulation. In nutrient-rich conditions, biofilms rapidly accumulate biomass until local resources deplete or wastes accumulate. Nutrient starvation can activate *agr*, leading to production of the proteases and surfactant peptides that break down the biofilm matrix [3]. In fact, reactivation of the *agr* system in an established *S. aureus* biofilm, by adding its autoinducing peptide (AIP) or by glucose exhaustion, triggers a rapid detachment of biofilm cells [3]. Thus, both shear forces and nutrient levels interact to determine not only the rate of biomass accumulation but also the timing and severity of dispersal and subsequent regrowth.

Despite the biological understanding of these processes, quantitative modeling frameworks capable of predicting the complete biofilm lifecycle (growth, dispersal (exodus), and regrowth) across environmental conditions remain limited. Existing approaches typically examine adhesion, growth, or dispersal in isolation, or treat biofilm development as a continuous, monotonic process, while more detailed simulations often require computationally intensive fluid–structure models that are not readily generalizable across conditions [7]. As a result, there is a lack of phase-resolved, predictive models that can quantitatively reconstruct and forecast biofilm behavior across coupled shear and nutrient environments. In this study, we address this limitation by developing a phase-resolved, interpolation-based kinetic framework for the *S. aureus* biofilm lifecycle under hydrodynamic conditions.

This study examines *S. aureus* biofilm development under a range of shear stresses and nutrient levels and analyzes the data with advanced computational modeling. A microfluidic flow system is used to grow *S. aureus* biofilms under controlled shear rates and nutrient concentrations, capturing time-lapse images of biofilm growth and dispersal. Notably, a characteristic “exodus” event is observed in many conditions, reflecting a sudden drop in biofilm coverage and followed in some cases by regrowth. To quantitatively capture these complex, non-linear dynamics, this analysis adopts an approach that partitions the coverage time-course into three phases (growth, exodus, regrowth) and fits each with an appropriate parametric model. Logistic functions have been used to describe saturating bacterial growth, and exponential functions naturally model detachment and regrowth kinetics. By extracting characteristic parameters for each phase, this study aims to create “life-cycle signatures” of biofilm behavior under each shear-nutrient condition. Data-driven modeling has become increasingly valuable in biomedical research for interpreting complex datasets [8]. Expanding on these findings, the study combines microfluidic experiments with a novel MATLAB (R2024a, The MathWorks, Inc., Natick, MA, USA)-based computational framework to quantify and model *S. aureus* biofilm development under combinations of shear rate and nutrient concentration. The study further introduces an automated phase-detection algorithm to identify *exodus* and *regrowth* transitions, followed by mechanistically interpretable, hierarchical kinetic models for each phase, enabling prediction of biofilm behavior at arbitrary intermediate environmental conditions. This integrative experimental and computational approach provides a quantitative representation of the *S. aureus* biofilm lifecycle and reveals how shear and nutrient environments jointly shape growth, dispersal timing, and regrowth potential.

## 2. Materials and Methods

### 2.1. Bacterial Strain and Culture Conditions

*S. aureus* Philips strain, previously isolated from a patient with osteomyelitis, was used throughout the study. This strain has been widely employed in studies of collagen adhesion and shear-responsive biofilm physiology [1,5,6,9]. Bacterial cultures were maintained as frozen stocks at −80 °C. Prior to experiments, 10 µL of stock culture was inoculated into 50 mL of Tryptic Soy Broth (TSB) in a 250 mL Erlenmeyer flask and incubated overnight at 37 °C with shaking at 200 rpm. Cultures were grown for 16–18 h to late-exponential phase, and optical density at 600 nm (OD_600_) was measured using a Thermo Scientific Genesys 20 spectrophotometer (Thermo Fisher Scientific, Waltham, MA, USA). The culture was then diluted in sterile phosphate-buffered saline (PBS) to a final concentration of approximately 1 × 10^8^ CFU/mL—as determined with a cell counter (Beckman Coulter Multisizer 4, Indianapolis, IN, USA).

### 2.2. Preparation of Growth Media

Tryptic Soy Broth without dextrose (TSB; Bacto^®^, BD, Franklin Lakes, NJ, USA) was used as the nutrient medium for all experiments. A 1× stock solution was prepared by dissolving 30 g of TSB powder in 1 L of deionized water, followed by autoclaving at 121 °C for 15 min to ensure sterility. The stock solution was used to prepare media of varying nutrient concentrations. To obtain lower concentrations, the 1× TSB stock was diluted with deionized water to yield 0.5× TSB (15 g/L) and 0.1× TSB (3 g/L). For high-nutrient conditions, a 2× TSB solution (60 g/L) was prepared by doubling the amount of TSB powder in the same volume of water prior to autoclaving. Throughout the preparation and handling process, aseptic techniques were employed to maintain sterility.

### 2.3. Biofilm Formation Assays and Time-Lapse Quantification

Biofilm experiments using the BioFlux 200 microfluidic system (Cell Microsystems, Durham, NC, USA) at 37 °C followed previously described methods [10,11,12,13], with minor modifications. Bacterial adhesion and growth were observed under hydrodynamic shear stress at four different shear rates: 10, 50, 100, and 300 s^−1^. The system was primed by flowing warm 1× TSB through the inlet wells at 1 dyne/cm^2^ for 5 min. Next, 20 µL of the bacterial suspension was introduced through the outlet wells at 1 dyne/cm^2^ for 10 s to seed the channels. The system was then left undisturbed for 20 min to allow for bacterial adhesion. Following adhesion, 1 mL of the appropriate TSB concentration was added to the inlet wells, and flow was initiated based on the desired shear rate. Shear rate values were controlled using the BioFlux software (version 2.6.0.9) interface.

Phase-contrast images were acquired every 30 min over a 10-h period using a Zeiss Axio Observer inverted microscope (Carl Zeiss Microscopy GmbH, Jena, Germany) integrated with the BioFlux 200 microfluidic flow system. Experiments were performed at 37 °C under constant shear conditions specific to each experimental setup. For each shear rate and nutrient concentration combination, three independent microfluidic channels were imaged in parallel, and the entire experiment was repeated across three biological replicates, yielding a total of nine measurements per condition.

Quantification of biofilm surface coverage was performed using ImageJ (version 1.54g, National Institutes of Health, Bethesda, MD, USA) following previously described workflows [14,15,16], with minor modifications. Surface coverage was defined as the percentage of the image area occupied by adherent bacterial cells. For each timepoint, values from the three channels were averaged to obtain one data point per experiment. The final dataset for each condition was generated by averaging results across the three biological replicates. All data were compiled and organized in tabular form for further analysis.

Only experiments that maintained stable hydrodynamic flow conditions and produced complete time-resolved surface coverage datasets over the full 10-h imaging period were included in the analysis. Microfluidic channels exhibiting flow disruption, imaging artifacts, or incomplete time series were excluded from downstream modeling and analysis.

### 2.4. Computational Modeling

Computational analyses were performed on the time-lapse biofilm data to identify key developmental transitions and to model growth dynamics under varying experimental conditions. All analyses used custom MATLAB scripts for data processing and model implementation (see Supplementary Information for full code and workflow details). For each combination of shear rate and TSB concentration, the biofilm time-series were averaged to generate representative surface-coverage trajectories, reducing noise and providing consistent inputs for downstream modeling.

As described in more detail below, the modeling then focused on automatically detecting the onset of the biofilm exodus phase and any subsequent regrowth within each averaged time-series. These detected transitions were used to segment the data, enabling phase-specific characterization and modeling of the initial growth, exodus, and, when present, regrowth dynamics.

### 2.5. Automated Exodus and Regrowth Phase Detection

To segment biofilm development into biologically meaningful phases, an automated algorithm was implemented in MATLAB to detect the onset of the exodus and regrowth phases based on characteristic changes in surface coverage over time. Exodus is described as an early dispersal phase observed in *S. aureus* biofilms after initiation [17]. Biologically, the exodus stage is marked by a sudden, significant reduction in biofilm biomass as a subpopulation of cells disperses from the confluent biofilm matrix [17,18]. To identify this event computationally, the analysis scanned each mean coverage time-course for a pronounced drop in biofilm coverage. This phase was defined as the first local maximum in the surface coverage curve followed by a sustained drop of at least 20% from the preceding maximum, evaluated over the immediately succeeding five time points. This threshold was selected to reflect biologically significant biomass dispersal while minimizing false positives due to minor fluctuations. The resulting time point was recorded as the beginning of the exodus phase ($t_{exodus}$).

Exodus detection was triggered when the algorithm encountered a local maximum, a point where *C*(*i*) *> C*(*i* − 1) and *C*(*i*) *> C*(*i* + 1) followed by a sustained drop of at least 20% in the subsequent 2.5-h period (five timepoints). This ensures that only abrupt, biologically meaningful biomass losses are labeled as dispersal events rather than minor fluctuations or noise. Similarly, regrowth is detected only after an exodus event and requires a local minimum followed by a ≥20% increase in coverage within the following 2 h (four timepoints). All timepoints are then automatically assigned to one of three stages: “Growth,” “Exodus,” or “Regrowth,” and the detected transition times (t_exodus_ and t_regrowth_) are exported as a summary table.

Following the identified exodus time, the algorithm evaluated subsequent time points for regrowth detection. Regrowth onset was defined as the first time point at which coverage increased by at least 20% from the minimum value observed during exodus, consistent with renewed accumulation of biomass. The regrowth time ($t_{regrowth}$) was recorded only when a true local valley was followed by a ≥20% increase in surface coverage within the next up to four time points, ensuring that the identified rise reflected genuine biological recovery rather than transient fluctuations. This automated criterion enabled consistent segmentation of each trajectory into three distinct intervals which can be separately modeled.

### 2.6. Logistic Model for Growth Phase

Early biofilm colonization typically follows rapid population increase that slows as space and nutrients become limiting, yielding an S-shaped (sigmoidal) curve. This behavior is well captured by the Verhulst (logistic) equation [19,20,21].

The initial accumulation phase was modeled using a three-parameter logistic function:

$$C_{growth}\left(t\right)=\frac{K}{1+e^{\left[-r_{growth}\left(t-t_{0}\right)\right]}},$$

where $C_{growth}\left(t\right)$ denotes biofilm surface coverage at time t, K is the carrying capacity (maximum attainable coverage), $r_{growth}$ is the intrinsic growth rate, and $t_{0}$ is the inflection time corresponding to the maximum growth rate. This function reflects classic population dynamics where growth begins exponentially but slows due to spatial constraints and nutrient depletion. Previous works have incorporated a logistic growth term into their biofilm flocculation model to represent biomass accumulation, noting that logistic dynamics appropriately capture growth that accelerates initially but slows as it approaches a system’s carrying capacity [22,23]. Parameters were estimated by fitting the model to the pre-exodus segment of each time-series using MATLAB’s nonlinear least squares optimization (nlinfit), minimizing the sum of squared errors between the model and observed data.

### 2.7. Exponential Model for Exodus Phase

In the exodus phase, biofilm biomass is lost as it detaches from the surface. This phenomenon is well described by first-order kinetics, and an exponential decay model is commonly used to represent it. Previous works have found that applying shear stress causes biofilm thickness and mass to decline exponentially and asymptotically [24]. In practice, simple exponential decay describes detachment behavior across multiple contexts, including shear-induced erosion and enzyme-mediated dispersal, and is widely used in biofilm modeling frameworks [3,25,26]. Thus, the exodus phase, characterized by rapid biomass loss, was modeled using an exponential decay function with a residual coverage plateau:

$$C_{exodus}\left(t\right)=A\;e^{-\lambda \left(t-t_{exodus}\right)}+C_{\mathrm{res}},$$

where A is the initial amplitude of biomass loss, λ is the exponential decay rate, $t_{exodus}$ is the exodus onset time, and $C_{\mathrm{res}}$ represents the asymptotic surface coverage after detachment. Parameters were fit to the coverage data spanning from $t_{exodus}$ to the regrowth onset $t_{regrowth}$ using nlinfit function in MATLAB. In cases where no regrowth occurred, the fitting interval extended to the end of the experiment.

### 2.8. Exponential Model for Regrowth Phase

After dispersal, the remaining biofilm biomass and incoming planktonic cells can regrow rapidly in a renewed colonization phase [27]. When a regrowth phase was detected, the post-exodus increase in coverage was modeled using an exponential rise toward a new plateau:

$$C_{regrowth}\left(t\right)=C_{\infty }-B\;e^{-\mu \left(t-t_{regrowth}\right)},$$

where $C_{\infty }$ is the new steady-state coverage after regrowth, B is the initial offset from $C_{\infty }$, μ is the regrowth rate constant, and $t_{regrowth}$ is the regrowth onset time. This function describes an accumulation process and approximates early-stage logistic behavior when growth resumes from a lower biomass state. Fitting was performed on data from $t_{regrowth}$ to the end of the experimental window using a custom nonlinear least-squares fitting procedure implemented in MATLAB, optimized via the fminsearch algorithm.

### 2.9. Interpolation to Predict Biofilm Kinetic Parameters and Lifecycle Dynamics

The computational framework was designed to enable the prediction of biofilm behavior across the experimental condition space. Model parameters from all fitted trajectories were organized into a lifecycle matrix indexed by shear rate and nutrient concentration. Each parameter, such as K, r, λ, $C_{\infty }$, and μ, was interpolated using MATLAB’s scatteredInterpolant function. This approach constructs smooth, continuous surfaces over irregularly spaced shear–nutrient data points, allowing model parameters to be estimated for intermediate conditions. In practice, interpolated parameter values were used to generate simulated coverage curves based on the same three-phase structure. This framework supports condition-specific forecasting of biofilm lifecycle dynamics and provides insight into how fluid shear and nutrient availability modulate growth, dispersal, and regrowth behavior across a continuous space. This follows standard practices in systems modeling: kinetic parameters are fitted at measured conditions, and spline interpolation is applied to infer parameter values between them [28,29]. This strategy aligns with approaches commonly used in environmental and microbial modeling to project experimentally derived rates onto a continuous condition space.

### 2.10. Statistical Analysis

Each phase-specific model was evaluated statistically using the coefficient of determination (R^2^) and root-mean-square error (RMSE). These metrics were computed for each fitted segment to assess how well the models described the observed data. Residuals were examined to confirm model assumptions and identify potential systematic deviations. Fits were performed on average coverage time-series from three biological replicates per condition. Mean values and standard deviations (±SD) were reported where applicable.

Statistical analyses served to evaluate the model performance and data reproducibility rather than to test specific biological hypotheses. Accordingly, all reported statistical measures are descriptive in nature, and no formal inferential hypothesis testing was performed. Conditions yielding low R^2^ values or unstable parameter estimates were interpreted cautiously and discussed qualitatively rather than used to draw strong quantitative conclusions. All statistical analyses and curve fitting were conducted in MATLAB.

## 3. Results

### 3.1. Microscopy and Surface Coverage Reveal Characteristic Biofilm Growth Patterns Under Flow

**Figure 1:**
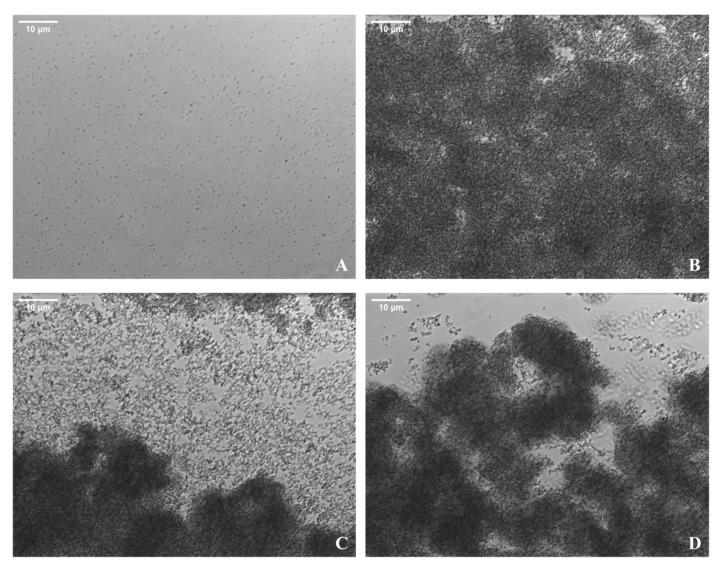
*S. aureus* biofilm development under flow at 100 s^−1^ and 1× TSB. Representative phase-contrast images of an *S. aureus* biofilm grown in the BioFlux 200 microfluidic system at a shear rate of 100 s^−1^ in 1× TSB at 37 °C. (**A**) Initial attachment and sparse microcolony formation at the start of the experiment. (**B**) Progression to a near-confluent biofilm 4.5 h into the experiment. (**C**) Observation of an exodus event 5.5 h into the experiment. (**D**) Subsequent biofilm regrowth 7.5 h into the experiment. Corresponding quantitative surface-coverage data are shown in Figure 2.

**Figure 2:**
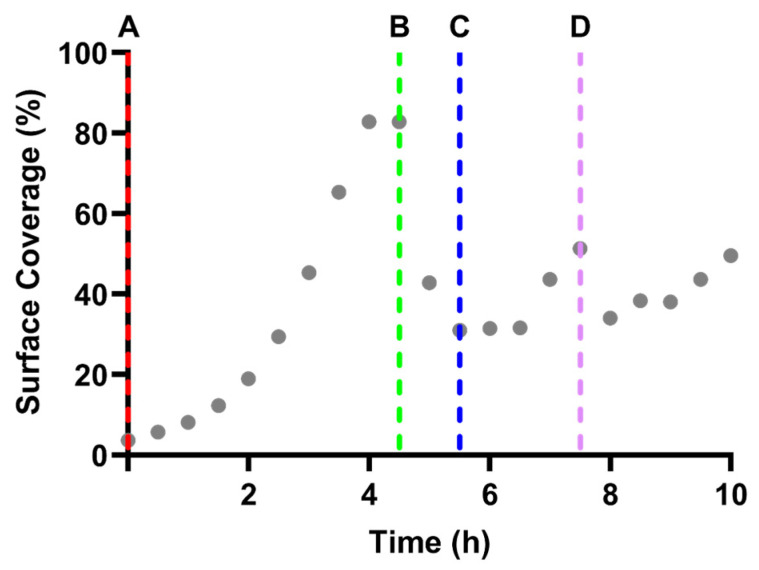
Quantitative surface-coverage dynamics corresponding to the microscopy sequence in Figure 1. Time-resolved surface-coverage profile of *S. aureus* biofilms grown at a shear rate of 100 s^−1^ in 1× TSB, corresponding directly to the morphological stages shown in Figure 1A–D. Grey markers show surface coverage (%) measured from phase-contrast images, while the vertical dashed lines labeled (**A**–**D**) indicate the time points corresponding to the images in Figure 1A–D, linking biofilm structural changes to quantitative coverage data.

Experimental assays revealed characteristic biofilm growth curves across all shear and nutrient conditions. Phase-contrast microscopy revealed progressive surface colonization which evolved into a confluent biofilm, followed by signs of partial detachment and regrowth (Figure 1). Quantitative surface coverage analysis confirmed a sigmoidal growth trajectory, with rapid accumulation of biomass reaching a peak coverage of ~82% by approximately 6 h (Figure 2B). This was followed by a sharp decline, characteristic of the biofilm “exodus” phase, during which coverage dropped to ~60% (Figure 2C). A moderate regrowth phase ensued, with surface coverage increasing slightly by the end of the experiment (Figure 2D).

Together, these data demonstrate a characteristic three-phase lifecycle: an initial logistic growth phase, a rapid biomass-loss event marking the exodus phase, and a subsequent period of partial recovery corresponding to regrowth. Structural changes observed microscopically aligned closely with quantitative shifts in surface colonization, highlighting consistent relationships between observed morphology and surface-coverage dynamics.

These figures provide a representative example of the data acquisition and analysis procedures used across all experimental runs, demonstrating how live imaging was integrated with computational surface quantification to support the evaluation of biofilm dynamics.

**Figure 3:**
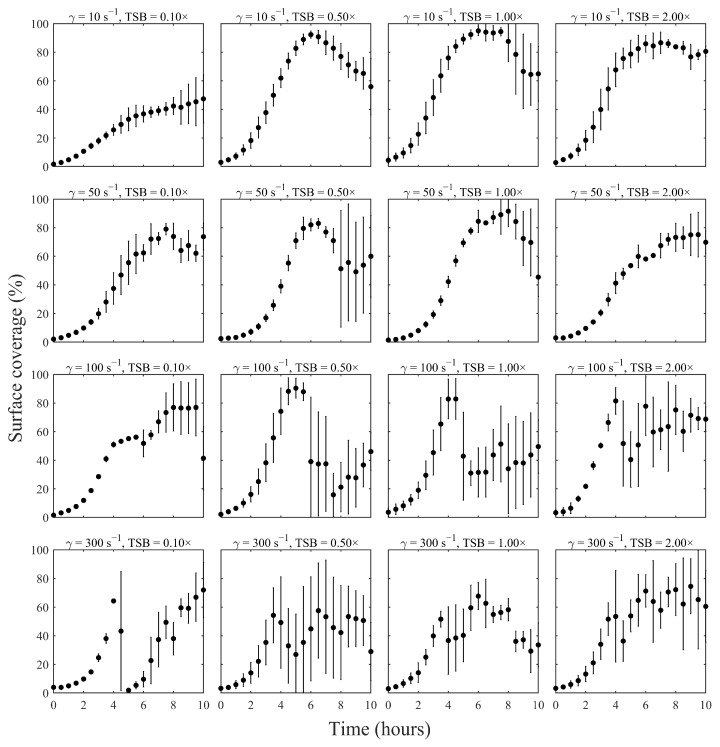
Biofilm surface-coverage trajectories for all 16 combinations of shear rate (10, 50, 100, 300 s^−1^) and TSB concentration (0.1×, 0.5×, 1×, 2×). Each panel shows the mean surface coverage (%) from three biological replicates, with error bars indicating standard deviation across replicates.

Despite differences in growth rate and maximum biomass, all biofilm experiments revealed a characteristic three-phase lifecycle: an initial logistic growth phase, a rapid biomass loss event marking the exodus phase, and a subsequent period of partial recovery during the final hours of the experiment, corresponding to regrowth (Figure 1, Figure 2 and Figure 3). Collectively, the results show how the structural progression observed microscopically aligns with quantitative changes in surface colonization under continuous shear flow (Figure 3).

Figure 3 summarizes biofilm surface coverage over time across all 16 shear and nutrient combinations. At the lowest shear rate (10 s^−1^), moderate to rich nutrient conditions (0.5–2× TSB) supported rapid colonization and extensive coverage, with biofilms reaching near saturation (~90%) within 6 h. In contrast, when nutrients were limited (0.1× TSB) but shear conditions remained constant, biofilm growth proceeded more slowly, reaching a plateau at roughly 50% surface coverage within 10 h.

At intermediate shear (50 s^−1^), a similar sigmoidal pattern emerged, with biofilms under moderate nutrient concentrations (0.5–1× TSB) achieving high surface coverage (~80–90%) by mid-exponential phase (7–8 h). Interestingly, doubling the nutrient concentration to 2× TSB did not increase peak biomass; instead, the maximum surface coverage was slightly lower (~75%) and occurred later (~9–10 h), suggesting diminishing returns or an earlier onset of dispersal at higher nutrient levels. These results indicate that nutrient enrichment beyond a moderate threshold does not necessarily promote greater biofilm accumulation under certain shear conditions.

At higher shear rates (100–300 s^−1^), biofilm development was increasingly restricted. Even with 1×- and 2×-TSB, peak surface coverage was reduced to ~60–80%, and these peaks occurred earlier, often within 4–6 h at 100 s^−1^. Under the highest shear conditions (300 s^−1^), biofilm coverage never exceeded 75%, and in nutrient-poor environments, some biofilms failed to exhibit a clear plateau at all, showing only gradual and limited surface colonization throughout the experiment. Together, these results demonstrate that intense hydrodynamic forces substantially limit biofilm development.

In summary, while shear stress and nutrient availability significantly modulated the rate and extent of biofilm accumulation, the underlying developmental pattern was conserved. All conditions displayed a single growth peak preceded by exponential expansion and followed by either a stable plateau or partial dispersal. This consistent one-peak trajectory, observed across the full matrix of environmental conditions, reflects a robust biofilm lifecycle shaped by both mechanical and metabolic constraints.

### 3.2. Automated Analysis Identifies Key Transition Points in the Biofilm Lifecycle

To systematically segment each biofilm growth curve into biologically interpretable phases, a MATLAB-based detection algorithm was used that identifies the exodus and regrowth events directly from the time-series surface coverage data. The algorithm identifies local extrema by scanning each curve and confirming the extrema through analysis of the curve over a short subsequent window.

**Figure 4:**
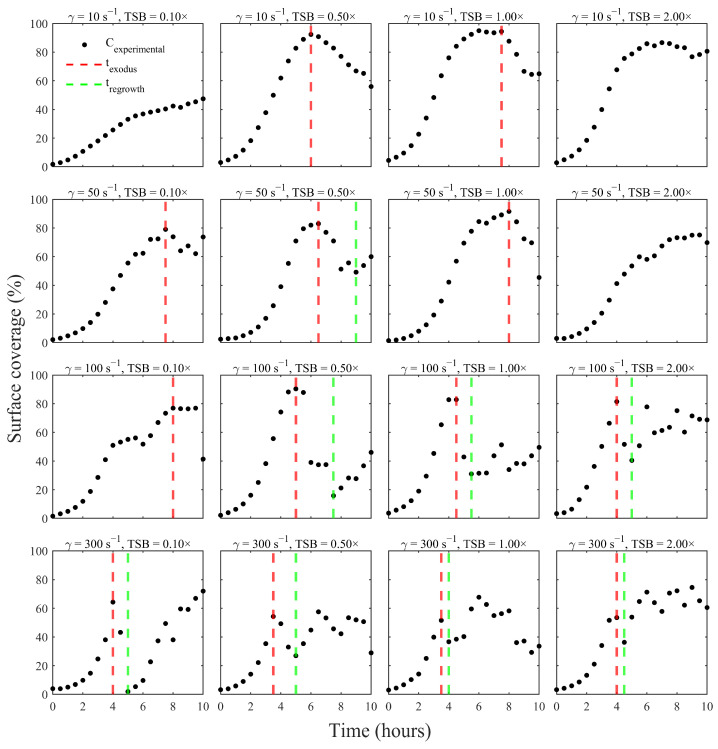
Phase segmentation of *S. aureus* biofilm lifecycle by the automated MATLAB algorithm across all 16 shear-nutrient conditions. Each panel shows the experimental surface-coverage curve (black markers) for a given shear rate and TSB concentration, overlaid with detected transition times. Red dashed vertical lines indicate the onset of the exodus phase (t_exodus_), and the green dashed vertical lines indicate the onset of regrowth (t_regrowth_) Across the matrix, exodus events were detected in 13 out of 16 conditions, and regrowth was observed in 8 of these, with earlier and more consistent exodus timing at higher shear rates (100–300 s^−1^).

Phase detection across all 16 shear-nutrient conditions is presented (Figure 4). The algorithm identified a valid exodus event (t_exodus_) in 13 out of 16 conditions, consistent with the expectation that *S. aureus* biofilms typically experience at least one pronounced detachment event during development under fluid flow conditions. Of these 13 cases, 8 exhibited a subsequent regrowth phase (t_regrowth_), where coverage recovered sufficiently after the minimum to satisfy the ≥20% rebound criterion. In the three conditions where no exodus event was detected, the surface coverage either increased steadily or plateaued with only minor fluctuations, and the model accurately classified the entire period as sustained growth.

The timing and presence of dispersal phases displayed clear dependence on shear rate and nutrient concentration. At higher shear rates (100–300 s^−1^), the algorithm frequently detected earlier and more consistent exodus events, reflecting the strong influence of hydrodynamic stress on detachment. For example, every condition at 300 s^−1^ exhibited a sharp peak followed immediately by a loss in coverage that met the drop threshold. Conversely, at 10 s^−1^, several conditions, notably at 0.1× and 2.0× TSB, never reached a local maximum followed by the required ≥20% decline, leading the algorithm to conclude that no dispersal event occurred within the 10-h window. Nutrient availability also shaped the observed dynamics. Environmental conditions with substantial biomass accumulation generally showed more prominent exodus signatures, as the large local maxima created clear biomass shedding that met the ≥20% criterion. For instance, at 10 s^−1^ and 0.50× TSB, the surface coverage rose above 90% before the algorithm detected a sharp biomass loss during the subsequent hour. In contrast, nutrient-poor conditions (0.10× TSB) often resulted in lower coverage numbers and less abrupt shedding of biomass.

After an exodus event, many affected biofilms entered a regrowth phase in which the remaining attached cells resumed proliferation; however, coverage never returned to its initial peak. For instance, under a shear of 100 s^−1^ with 1× TSB, biofilm coverage dropped from ~82% at its peak to ~60% during the exodus, then gradually rebounded to ~70% by the experiment’s end. By contrast, under the most extreme shear stress of 300 s^−1^, only a modest or negligible regrowth was observed after dispersal. Presumably because the intense flow continually impeded the re-establishment of biofilm biomass. In moderate-shear environments, however, biofilms sometimes exhibited a pronounced second growth phase after the exodus event.

Overall, the refined MATLAB algorithm provides a reproducible and parameterized way to detect biofilm lifecycle transitions across all experimental conditions. By enforcing drop- and rise-based thresholds on validated local extrema, the method robustly separates growth, exodus, and regrowth phases and yields a standardized dataset suitable for downstream quantitative modeling. The patterns summarized in Figure 4 illustrate the interplay of shear, nutrient concentration, and temporal biofilm dynamics, revealing how environmental conditions govern the initiation and strength of dispersal and regrowth events in *S. aureus*.

### 3.3. Three-Phase Kinetic Fits Quantify Biofilm Development Under Flow

To quantify how *S. aureus* biofilms progressed through the three lifecycle phases, the phase-specific kinetic models described earlier (logistic growth, exponential exodus, exponential regrowth) were fitted to the mean surface-coverage trajectories for each shear-nutrient combination. Fits were performed only on the time windows labeled as “Growth,” “Exodus,” or “Regrowth” by the automated phase-detection algorithm. This ensured that each model was constrained to the specific data segment it was intended to represent. All fitted parameters obtained from the logistic growth, exponential exodus, and regrowth models are summarized in Table 1, Table 2 and Table 3. These parameter values form the basis of the phase model fit and the lifecycle model reconstruction shown in subsequent sections and serve as the inputs to the interpolation-based prediction framework.

#### 3.3.1. Growth Phase

**Figure 5:**
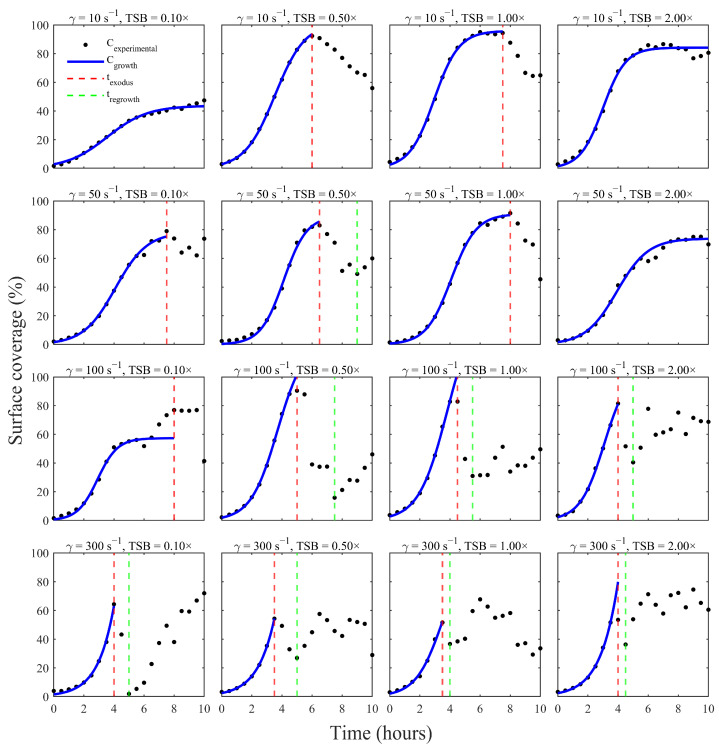
Logistic model fit for the growth phase of *S. aureus* biofilms across all shear-nutrient combinations are shown here. For each condition, experimental mean surface coverage (black markers) prior to the detected exodus time is fit with a three-parameter logistic function (blue curves), representing the carrying capacity, growth rate, and inflection time.

In most conditions, the logistic model closely captures the initial lag, rapid accumulation, and approach to a plateau, with high coefficients of determination (median R^2^ ≈ 0.996) and low RMSE (Figure 5). These fits provide phase-specific parameters that summarize growth capacity and kinetics for each environmental condition. Visually, the model closely followed the initial lag, rapid accumulation, and approach to a plateau in most conditions, including those with very high coverage at low shear (e.g., 10–50 s^−1^, 0.5–1× TSB) and those with lower biomass at high shear (100–300 s^−1^). Quantitatively, the logistic model achieved excellent performance across the matrix (mean R^2^ ≈ 0.976 and median R^2^ ≈ 0.996, ranging from 0.78–0.99; mean RMSE ≈ 2.62 and median RMSE ≈ 1.45 coverage units). The few weaker fits corresponded to conditions where the experimental curve did not exhibit a clean sigmoidal shape, for example, at 300 s^−1^ and 2.0× TSB, where coverage remained low and noisy or showed only partial saturation.

The fitted growth parameters (Table 1) behaved consistently with the qualitative trends observed in Figure 3. The carrying capacity, K, tracked the observed peak coverage for each condition, with larger plateaus under low shear and moderate nutrients and smaller plateaus when shear was strong or nutrients limiting. The growth-rate parameter $r_{growth}$ was generally higher in conditions that produced rapid early colonization (e.g., 10–50 s^−1^, 0.5–1× TSB) and lower in curves that rose slowly or never clearly plateaued. The inflection time $t_{0}\;$shifted earlier at higher shear and higher nutrient levels, capturing the observation that biofilms in these environments reached their maximum coverage sooner, even when that maximum was modest. In some cases, the fitted carrying capacity K exceeded 100%, when the growth phase did not reach a clear plateau within the experimental window. Thus, leaving the logistic asymptote weakly constrained and dominated by model extrapolation.

#### 3.3.2. Exodus Phase

**Figure 6:**
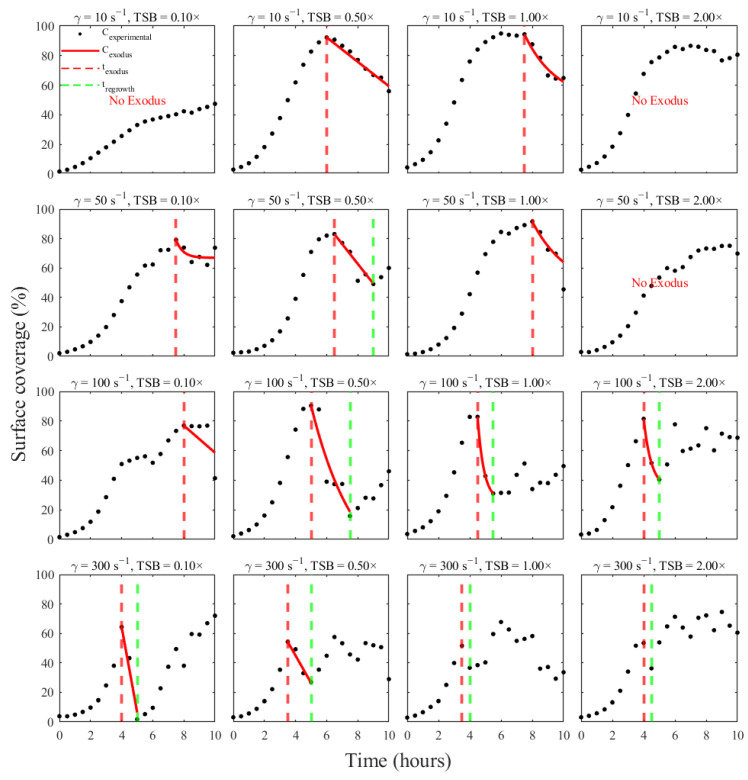
Exponential decay model fits to the exodus (dispersal) phase of *S. aureus* biofilms. For each condition in which an exodus event was detected, the coverage time-series between t_exodus_ and either t_regrowth_ or the end of the experiment is modeled with an exponential decay function (red curves). Black markers represent experimental mean surface coverage over the exodus interval.

The model reproduced the sharp, monotonic drops in surface coverage in most conditions where an exodus event was detected (Figure 6). The model captures the abrupt, monotonic loss of biomass in many conditions, yielding generally strong agreement (median R^2^ ≈ 0.95). Across these conditions, the exodus model showed relatively strong agreement with data (mean R^2^ ≈ 0.84 and median R^2^ ≈ 0.95, range 0.40–0.97; mean RMSE ≈ 5.6 and median RMSE ≈ 4.0). High-quality fits were obtained for classic exodus profiles such as 10 s^−1^, 0.5–1× TSB and 50–100 s^−1^, 0.5–1× TSB, where coverage peaked and then declined steadily over several time points (Figure 6).

The fitted onset coverage A closely matched the observed peak at the start of the exodus window, while the residual plateau $C_{\mathrm{res}}$ captured the remaining attached biomass after detachment. The decay rate λ increased in conditions with rapid loss of biomass (e.g., 300 s^−1^, 0.1–0.5× TSB), consistent with strong shear-driven detachment, and was smaller in conditions where coverage declined more gradually. Importantly, the exodus model could not be reliably fit in all cases. At 300 s^−1^ in 1× and 2× TSB, the exodus phase was extremely short, spanning essentially a single 30-min interval between peak and trough. This provided too few points for a stable exponential fit, resulting in undefined estimates. Negative values of the residual coverage parameter $C_{res}$ appeared in some exodus fits, particularly under high shear where detachment event was short-lived. These values arose from applying an exponential decay model to sparsely sampled exodus dynamics and reflecting limited parameter identifiability rather than negative biofilm coverage. These failures later propagate into the lifecycle model, where the exodus segment is effectively absent for those conditions.

#### 3.3.3. Regrowth Phase

**Figure 7:**
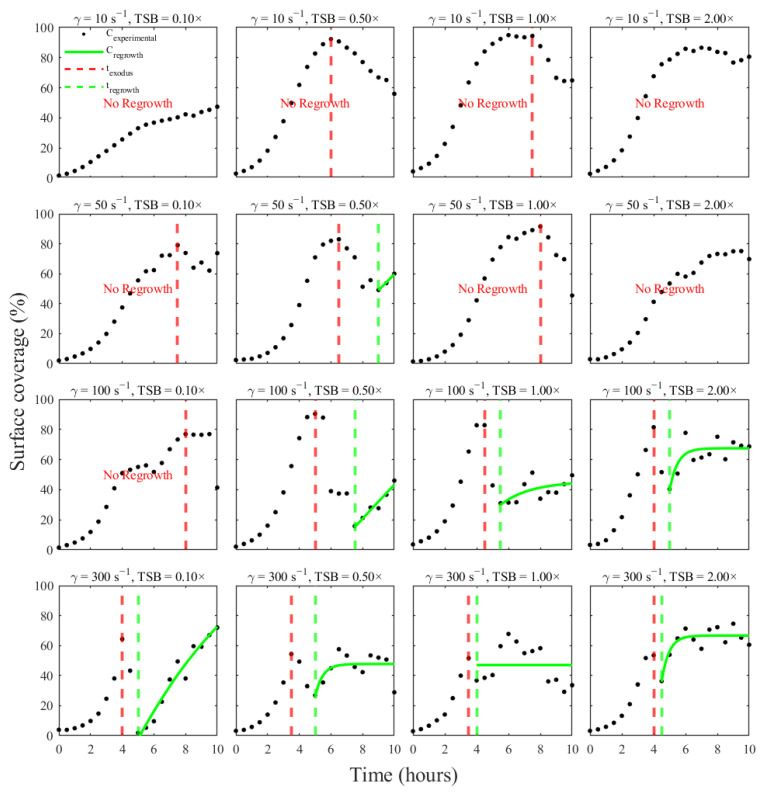
Regrowth-phase fits for conditions showing a post-exodus increase in biofilm coverage. Panels display the time window after t_regrowth_ for each shear-nutrient combination in which the automated algorithm identified regrowth. Black markers indicate experimental mean surface coverage, and green curves show exponential rise functions fit to these regrowth segments.

Regrowth fits (green curves) are shown in Figure 7 for conditions where the algorithm detected a post-exodus increase in coverage. As expected, this phase was more heterogeneous than growth or exodus. In some moderate-shear conditions (50 s^−1^, 0.5× TSB; 100 s^−1^, 1× TSB), the regrowth model closely reproduces the observed recovery toward a new plateau (R^2^ approaching 0.99). In contrast, certain high-shear conditions exhibit only weak or noisy changes after exodus, resulting in poorer fits and low explanatory power (300 s^−1^, 1× TSB). Overall, these regrowth fits provide phase-specific parameters describing the rate and extent of secondary biomass accumulation after dispersal, while highlighting the conditions under which regrowth is strongly suppressed.

Some conditions, such as 50 s^−1^, 0.5× TSB and 100 s^−1^, 1× TSB, exhibited clear recovery toward a new plateau and were well captured by the exponential rise model (Figure 7), with R^2^ values approaching 0.99 and low RMSE under 2.30. In contrast, several high-shear conditions showed only modest or noisy changes after exodus, resulting in weaker fits (overall mean R^2^ ≈ 0.73 median R^2^ ≈ 0.75; range 0–0.99; mean RMSE ≈ 5.60 and median RMSE ≈ 5.34).

The regrowth parameters reflected these behaviors (Table 3). The estimated post-regrowth plateau $C_{\infty }$ was consistently lower than the original K, indicating that biofilms rarely regained their pre-exodus coverage within the 10-h window. The regrowth rate constant μ tended to be larger in moderate shear regimes where biofilms partially recovered and smaller in regimes where continued flow suppressed re-colonization. In the most extreme case (300 s^−1^, 1× TSB), the regrowth model provided essentially no explanatory power (R^2^ ≈ 0), highlighting that the data in this condition did not follow a simple exponential recovery and that any fitted parameters in such cases should be interpreted with caution. Similarly in some regrowth conditions, the fitted asymptotic plateau $C_{\infty }$ exceeded 100%. This reflected extrapolation of the regrowth model beyond the observed data range when post-exodus recovery was weak or poorly resolved.

### 3.4. Integrated Three-Phase Models Reconstruct Biofilm Lifecycles Across Conditions

Finally, the interpolated parameter surfaces were used to reconstruct the entire biofilm lifecycle for each original shear-nutrient condition and to compare these composite predictions with the experimental data. For each condition, the corresponding values of K, $r_{growth}$, $t_{0}$, $t_{\mathrm{exodus}}$, A, $C_{\mathrm{res}}$, λ, $t_{regrowth}$, B, $C_{\infty }$, and μ were retrieved from the lifecycle parameter table using ‘scatteredInterpolant’ function in MATLAB. These parameters were then assembled into a single piecewise trajectory consisting of a logistic growth segment, an exponential detachment segment (when available), and an exponential regrowth segment (when available), as defined previously.

**Figure 8:**
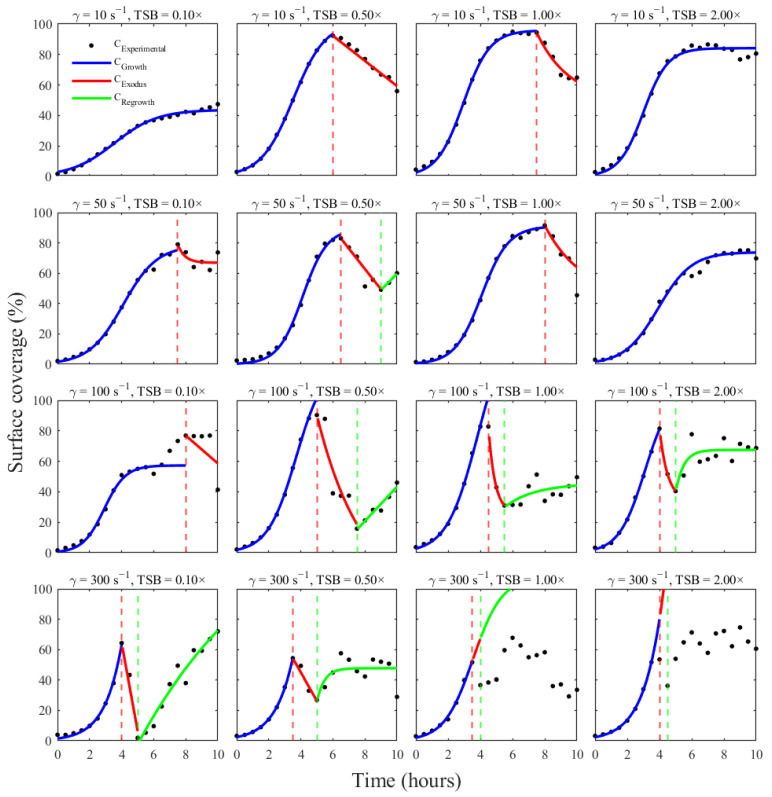
Piecewise reconstruction of the *S. aureus* biofilm lifecycle using phase-specific kinetic models across all 16 shear–nutrient combinations. The resulting three-phase lifecycle curves (blue = growth, red = exodus, green = regrowth) overlaid on the experimental surface-coverage data for all 16 conditions.

The composite lifecycles provide a concise, mechanistic summary of biofilm behavior under each environmental condition and form the basis for interpolation across the shear-nutrient space. In most cases, the composite model visually reproduced the key features of the observed dynamics: a sigmoidal rise to a peak, a subsequent drop during exodus, and partial recovery during regrowth (Figure 8). Agreement was particularly strong in low- to moderate-shear regimes (10–100 s^−1^) with intermediate nutrient levels, where each individual phase fit was well constrained. Here, the integrated three-phase model not only captured the timing of the transitions but also closely matched the magnitude of the peak and post-exodus plateau.

Deviations between the integrated model and data were largely confined to conditions where one or more phase-specific fits were poorly determined. For example, at 300 s^−1^ in 1× and 2× TSB, the exodus phase lasted only a single 30-min sampling interval, providing insufficient information for a robust exponential fit. In these cases, the exodus parameters were undefined, and the global predictor defaulted to extending the growth model across the nominal exodus window, effectively smoothing over the rapid drop observed experimentally. Similarly, in conditions where regrowth was weak or noisy (e.g., 300 s^−1^, 1× TSB), the regrowth segment of the model did not fully capture the irregular post-exodus behavior. These discrepancies highlight the sensitivity of the composite lifecycle model to short or poorly sampled phases and indicate where additional temporal resolution or replicate data would most improve model fidelity.

Despite these limitations, the three-phase model representation provides a coherent, mechanistic summary of biofilm development under flow. By mapping each time point to a specific kinetic regime and tying the regime to interpretable parameters (carrying capacity, detachment rate, regrowth rate, etc.), the model offers a compact way to compare biofilm lifecycles across shear and nutrient conditions and to predict how the system would behave at intermediate points in the experimental space.

## 4. Discussion

Biofilms in real systems can exhibit cyclical growth and dispersal behavior, as shown in previous work [30,31]. The three-phase model developed in this study can capture such cycles in a simple, parametric form. The study reveals biologically meaningful trends in how shear stress and nutrient availability shape the *S. aureus* biofilm life cycle. Under all tested conditions, biofilms exhibited a single growth peak followed by dispersal and, in many cases, partial regrowth (Figure 3). These patterns reflect a balance between improved nutrient delivery and shear-induced erosion. As postulated in previous work, high-flow conditions can cause biofilm biomass to rise briefly before declining due to shear-induced erosion, whereas gentler flow allows sustained biomass growth until nutrients are depleted [32]. Similarly, the timing and magnitude of the exodus phase depended jointly on shear and accumulated coverage (Figure 6). This observation echoes the nuclease-mediated ‘exodus’ stage reported for flow-grown *S. aureus* biofilms (~6 h), which precedes tower formation [27]. Robust exodus requires both sufficient biofilm mass and adequate shear, whereas low-biomass or low-shear conditions may bypass a distinct detachment stage.

From a biological perspective, the observed growth-exodus-regrowth dynamics are consistent with regulated dispersal mechanisms in *S. aureus*, where accumulated biomass and environmental stress trigger coordinated detachment. Prior studies have shown biofilm dispersal through matrix-degrading proteases and surfactant-like phenol-soluble modulins, which weaken cell–cell and cell-surface interactions and facilitate biomass release under stressful conditions [4,33,34]. The joint dependence of exodus timing and magnitude on shear stress and nutrient availability suggests that mechanical forces and metabolic state act together to govern dispersal decisions during biofilm development.

The three-phase kinetic model developed in this study represents a novel, MATLAB framework for capturing the full biofilm lifecycle under hydrodynamic conditions. Unlike prior studies that focus on individual stages of biofilm development or single outcome metrics [5,7,23,24], this framework enables direct, quantitative comparison and prediction of complete biofilm lifecycles across environmental conditions. Each phase’s duration and rate constants can be extracted from time-series data (Table 1, Table 2 and Table 3). This approach explicitly segments the biomass curve into biologically meaningful stages that yield directly interpretable parameters. Logistic growth captures the initial proliferative phase, while the exponential exodus term models the rapid biomass loss once detachment begins. The final exponential regrowth phase quantifies how quickly residual cells rebound after sloughing. This interpretability is valuable because, unlike complex computational models, each parameter maps directly onto a specific biological process (growth, exodus or regrowth), which could ultimately guide hypotheses about underlying mechanisms in clinical and biomedical-device contexts.

In vivo, *S. aureus* and other pathogens commonly form biofilms on implants (such as catheters and orthopedic devices) and on tissues [35]. Studies emphasize that biofilm formation on devices depends sensitively on material, flow and nutrient conditions [35,36]. As the biofilm matures under favorable conditions it can trigger host inflammation or device failure, and when shear or nutrient changes induce detachment, the released planktonic cells can seed secondary infections. Studies have estimated that ~65% of persistent infections are biofilm-related, and that detachment of cells from biofilms may explain the high rate of metastatic *S. aureus* infections seen clinically [6]. In this study, discrete experimental results are used to develop a continuous parameter surface across the shear and nutrient domains. This approach is essentially data-driven surface fitting, like Response Surface Methodology (RSM), which assumes that a multivariate response can be approximated by a smooth surface to predict outcomes at untested factor combinations [37]. By capturing biologically driven trends (e.g., faster biofilm growth with higher nutrients until shear stress becomes inhibitory), the interpolated surface provides continuous prediction across the experimental domain in a biologically plausible way. This means unmeasured scenarios within the tested range can be forecasted without additional experiments, leveraging the assumption that microbial responses change gradually rather than abruptly across environmental settings [37]. Predictive microbiology routinely employs such secondary models [38]. Clinically, these findings are relevant to device-associated infections, where biofilms experience defined flow and nutrient environments. The ability to predict when biofilms transition from stable growth to active dispersal may inform catheter management, surface design, or treatment timing to reduce the risk of bacterial dissemination and secondary infection.

Several studies demonstrate that interpolation-based frameworks are valid and powerful tools in biological modeling. In systems biology, a central goal is to build models that generalize beyond observed data to predict system dynamics under new conditions, a challenge that has been met in simpler microbial systems [39]. For instance, data-driven models of gene regulation in unicellular organisms have successfully anticipated behavior in untested conditions [39]. Modern modeling techniques demonstrate remarkable predictive reach. In one study, researchers trained a neural-network “metamodel” using only 128 discrete parameter combinations and successfully reproduced the outcomes of more than 10,000 previously untested parameter sets in a stochastic microbial network [40]. Such results illustrate that a carefully constructed parameter surface can capture the underlying biological relationships well enough to forecast complex dynamics far from the original data points. By honoring the continuous nature of environmental influences on biofilm processes, this approach serves as a robust, data-driven predictor for biofilm growth, dispersal (exodus), and regrowth trajectories across a continuum of conditions. The interpolated parameter surface thus functions as a forecasting tool that bridges sparse experimental data and the full lifecycle behavior of biofilms within the shear-nutrient landscape, in agreement with established predictive modeling practices [37,38].

This work represents a step toward a predictive understanding of biofilm-associated infection risks and control strategies in real-world settings. Once calibrated to empirical data, the computational framework could serve as a virtual testbed for identifying high-risk conditions and evaluating potential treatment schedules. Additionally, explicit parametric models further provide a foundation for future machine-learning tools. As more data are collected across conditions, ML methods (e.g., neural networks or Gaussian processes) could learn the mapping from shear and nutrient inputs directly to model parameters or biofilm states. This aligns with the growing interest in applying ML to biofilm prediction and control [41]. Combining ML with interpretable kinetic models is especially appealing because the kinetic framework could enable trained models to predict biofilm state with minimal experimental input. This would shift the field from reactive diagnosis toward proactive prediction.

Furthermore, this study highlights several opportunities for future improvements. Biofilm dynamics were quantified using two-dimensional surface coverages, providing a clear and accessible measure of spatial evolution; however future work could benefit from incorporating three-dimensional insights into the biofilm structure parameters including, biofilm thickness, porosity and matrix composition. Experiments were performed using a single *S. aureus* strain under controlled in vitro conditions and therefore do not account for strain-to-strain variability, polymicrobial communities, or host immune interactions. Additionally, the exponential representation of detachment and regrowth may oversimplify stochastic sloughing events observed in real biofilms. Predictions should therefore be interpreted within the tested shear–nutrient domain.

Taken together this work advances quantitative, predictive biofilm modeling in biomedical engineering. It shows that a simple, interpretable kinetic framework can capture key *S. aureus* biofilm lifecycle dynamics across flow and nutrient conditions, providing a tool for risk assessment, device design, and infection-control planning. Ultimately, shifting from descriptive characterization to predictive modeling may enable earlier warnings of biofilm outbreaks and more rational design of anti-biofilm materials.

## Figures and Tables

**Table 1:** Logistic growth parameters across shear-nutrient conditions. A logistic model was used to fit biofilm accumulation: $C_{\mathrm{growth}}(t)=\frac{K}{1+e^{\left[-r_{\mathrm{growth}}\left(t-t_{0}\right)\right]}}$. Parameters K, $r_{\mathrm{growth}}$, and $t_{0}$ are defined in Section 2. R^2^ and RMSE quantify goodness of fit.

Shear Rate	TSB Concentration	K (%)	r_growth_ (h^−1^)	t_0_ (h)	R^2^	RMSE
10 s^−1^	0.1×	38.46	1.07	6.02	0.99	1.66
	0.5×	81.55	1.25	3.71	0.99	2.50
	1.0×	95.42	1.19	3.38	0.99	2.05
	2.0×	87.57	0.79	4.18	0.99	1.34
50 s^−1^	0.1×	65.42	1.20	4.08	1.00	1.17
	0.5×	93.09	1.28	3.07	0.99	2.41
	1.0×	88.69	1.06	3.81	0.99	1.82
	2.0×	73.87	0.96	3.91	0.99	1.51
100 s^−1^	0.1×	49.40	0.96	4.33	0.99	1.50
	0.5×	68.69	0.91	4.25	0.99	1.22
	1.0×	152.39	1.16	3.70	0.96	5.33
	2.0×	87.83	0.88	3.93	0.99	1.40
300 s^−1^	0.1×	15,861.23	2.19	2.89	0.99	7.27
	0.5×	4271.34	1.96	2.91	0.99	8.32
	1.0×	104.58	1.72	2.65	0.90	6.48
	2.0×	2837.47	2.62	2.51	0.78	8.84

**Table 2:** Exponential exodus parameters across shear-nutrient conditions. The exodus phase was modeled using an exponential function: $C_{\mathrm{exodus}}(t)=A\;e^{-\lambda (t-t_{\mathrm{exodus}})}+C_{\mathrm{res}}.$ Parameters A, λ, $t_{\mathrm{exodus}}$, and $C_{\mathrm{res}}$ follow definitions provided in Section 2. Dashes indicate conditions where no exodus was detected.

Shear Rate	TSB Concentration	t_exodus_ (h)	A (%)	C_res_ (%)	λ (h^−1^)	R^2^	RMSE
10 s^−1^	0.1×	—	—	—	—	—	—
	0.5×	6.50	91.67	−5873.40	1.46	0.42	7.94
	1.0×	6.00	95.36	64.47	0.55	0.98	1.70
	2.0×	7.50	88.06	64.25	0.44	0.95	2.50
50 s^−1^	0.1×	6.00	69.51	66.35	0.31	0.55	2.70
	0.5×	6.50	92.11	−179.11	0.32	0.87	4.74
	1.0×	8.00	91.52	43.58	0.25	0.72	3.50
	2.0×	7.50	74.65	45.51	0.33	0.90	2.26
100 s^−1^	0.1×	6.50	52.39	−61.38	0.43	0.71	1.78
	0.5×	7.00	69.98	−27.81	0.37	0.87	2.02
	1.0×	6.50	81.89	58.79	0.33	0.93	2.54
	2.0×	6.00	79.16	61.72	0.28	0.97	1.92
300 s^−1^	0.1×	5.00	49.16	−95.56	0.43	0.93	2.09
	0.5×	5.50	75.13	−112.35	0.44	0.95	3.08
	1.0×	—	—	—	—	—	—
	2.0×	—	—	—	—	—	—

**Table 3:** Regrowth parameters across shear-nutrient conditions exhibiting post-exodus recovery. Regrowth was modeled using an exponential function: $C_{\mathrm{regrowth}}(t)=C_{\infty }-B\;e^{-\mu (t-t_{\mathrm{regrowth}})}.$ Parameter definitions (B, $C_{\infty }$, μ, $t_{\mathrm{regrowth}}$) are provided in Section 2. Dashes indicate conditions where no regrowth was detected.

Shear Rate	TSB Concentration	t_regrowth_ (h)	B (%)	C_∞_ (%)	μ (h^−1^)	R^2^	RMSE
10 s^−1^	0.1×	—	—	—	—	—	—
	0.5×	—	—	—	—	—	—
	1.0×	—	—	—	—	—	—
	2.0×	—	—	—	—	—	—
50 s^−1^	0.1×	—	—	—	—	—	—
	0.5×	9.00	48.89	467.93	0.03	0.99	0.38
	1.0×	—	—	—	—	—	—
	2.0×	—	—	—	—	—	—
100 s^−1^	0.1×	—	—	—	—	—	—
	0.5×	7.50	15.13	1081.26	0.01	0.95	2.29
	1.0×	5.50	29.90	45.56	0.53	0.40	5.52
	2.0×	5.00	39.52	67.50	2.00	0.61	6.47
300 s^−1^	0.1×	5.00	−2.23	170.87	0.11	0.95	5.15
	0.5×	5.00	25.79	47.69	2.10	0.42	7.51
	1.0×	4.00	47.00	47.00	0.22	0.00	12.54
	2.0×	4.50	35.83	66.68	2.16	0.75	4.93

## Data Availability

All raw data supporting the findings of this study are available from the corresponding author upon request. The MATLAB code and associated workflow are included in the Supplementary Information.

## Supplementary Materials

The following supporting information can be downloaded at https://www.mdpi.com/article/10.3390/pathogens15010118/s1: S1. MATLAB Code and Workflow.
