# Supplementary material for: A Predictive Computational Framework for Staphylococcus aureus Biofilm Growth Stages in Hydrodynamic Conditions
Source: Pathogens. 2026 Jan 21;15(1):118. doi: 10.3390/pathogens15010118 (PMC12844980; doi:10.3390/pathogens15010118)
Supplement: Supplementary file 1 [file pathogens-15-00118-s001.zip › pathogens-4086234-supplementary.pdf]

Supplementary Information

A Predictive Computational Framework for *Staphylococcus aureus* Biofilm Growth Stages in Hydrodynamic Conditions

Sarees Shaikh [1], Abiye Mekonnen [1], Abdul Nafay Saleem [2] and Patrick Ymele-Leki [1,*]

[1] Department of Chemical Engineering, Howard University, Washington, DC 20059;
[2] Department of Electrical Engineering and Computer Science, Howard University, Washington, DC 20059
* Correspondence: patrick.ymeleleki@howard.edu

S1. MATLAB Code and Workflow

This Supplementary Information provides the MATLAB code used to (i) detect growth, exodus, and regrowth phases in *Staphylococcus aureus* biofilm surface-coverage data, (ii) fit phase-specific kinetic models, and (iii) construct interpolated parameter surfaces for lifecycle prediction across shear-nutrient conditions. All codes operate on the experimental time-series dataset organized as Shear, Conc, Time, and Mean columns.

S1.1. Phase Detection and Timepoint Labelling: This script reads the surface coverage dataset (sc_v_time_data.csv), detects the first exodus and regrowth events for each shear-nutrient condition using local extrema with 20% drop or 20% rise criteria, labels all timepoints as growth, exodus, or regrowth, and generates a tiled figure of all conditions.

```
% Input CSV format:
%   Shear,Conc,Time,Mean
%
% Exodus criterion:
%   - Scan for the first local peak: y(i) > y(i-1) AND y(i) > y(i+1)
%   - Look at ANY of the next up-to-5 points (i+1 .. i+5, clipped at end).
                        %   - If min(futureVals) <= 0.8 * y(i)  (≥ 20% DROP), Exodus
                        at t(i).
                        %
% Regrowth criterion:
%   - After Exodus time, scan for the first local valley:
%       y(i) < y(i-1) AND y(i) < y(i+1)
%   - Look at ANY of the next up-to-4 points (i+1 .. i+4, clipped at end).
%   - If max(futureVals) >= 1.2 * y(i)  (≥ 20% RISE), Regrowth at t(i).

clear; clc;

% ---------- CONFIGURATION ----------
dataFile        = "sc_v_time_data.csv";   % CSV file name
```

```
shearColName    = 'Shear';
concColName     = 'Conc';
timeColName     = 'Time';
covColName      = 'Mean';

summaryOutFile  = "exodus_regrowth_summary_matlab.csv";
labeledOutFile  = "labeled_biofilm_data_matlab.csv";

% Global font
set(groot, 'DefaultAxesFontName', 'Times New Roman');
set(groot, 'DefaultTextFontName', 'Times New Roman');

% ---------- LOAD DATA ----------
T = readtable(dataFile);

requiredCols = {shearColName, concColName, timeColName, covColName};
varNames     = T.Properties.VariableNames;
assert(all(ismember(requiredCols, varNames)), ...
    'One or more expected column names not found in the CSV table.');

shearVals = unique(T.(shearColName));
concVals  = unique(T.(concColName));

summaryRows = {};
labeledRows = {};

% ---------- FIGURE & TILED LAYOUT ----------
fig = figure('Name','Phase detection by condition', ...
             'Position',[100 100 1200 900], ...
             'Color','w');

tl = tiledlayout(numel(shearVals), numel(concVals), ...
                 "TileSpacing","compact", "Padding","compact");

legendAdded = false;

for i = 1:numel(shearVals)
    G = shearVals(i);
    for j = 1:numel(concVals)
        N = concVals(j);

        % ---- Subset data for this shear-conc condition ----
        idx = (T.(shearColName) == G) & (T.(concColName) == N);
        Tsub = T(idx,:);
        if isempty(Tsub)
            nexttile; axis off;
            continue;
        end

        % Sort by time
        Tsub = sortrows(Tsub, timeColName);
        t = Tsub.(timeColName);
        y = Tsub.(covColName);

        % ---- Phase detection ----
```

```
[tExo, tReg] = detectExodusRegrowth_localExtrema(t, y);

% Store in summary
summaryRows(end+1, :) = {G, N, tExo, tReg};

% ---- Label each timepoint ----
for r = 1:numel(t)
    ti = t(r);

    if isnan(tExo)
        % No exodus → everything is Growth
        stage = "Growth";

    elseif isnan(tReg)
        % Exodus but NO regrowth:
        %   t < tExo    → Growth
        %   t >= tExo → Exodus
        if ti < tExo
            stage = "Growth";
        else
            stage = "Exodus";
        end
    else
        % Both exodus and regrowth detected
        if ti < tExo
            stage = "Growth";
        elseif ti < tReg
            stage = "Exodus";
        else
            stage = "Regrowth";
        end
    end

    labeledRows(end+1, :) = {ti, y(r), G, N, char(stage)}; %#ok<SAGROW>
end

% ---- Plot panel ----
ax = nexttile;
hold(ax,'on');

hData = plot(ax, t, y, 'ko', ...
    'MarkerFaceColor','k', ...
    'LineStyle','none', ...
    'MarkerSize',3);
hEx  = [];
hReg = [];
if ~isnan(tExo)
    hEx = xline(ax, tExo, 'r--', 'LineWidth', 2.5);
end
if ~isnan(tReg)
    hReg = xline(ax, tReg, 'g--', 'LineWidth', 2.5);
end
xlim(ax, [0 10]);
xticks(ax, [0 2 4 6 8 10]);
ylim(ax, [0 100]);
```

```
        yticks(ax, [0 20 40 60 80 100]);
        box(ax,'on');
        ax.XGrid = 'off';
        ax.YGrid = 'off';

        title(ax, sprintf('\\gamma = %g s^{-1}, TSB = %.2fx', G, N));

        isLeftColumn = (j == 1);
        isBottomRow  = (i == numel(shearVals));

        if isLeftColumn
            ax.YTickLabelMode = 'auto';
        else
            ax.YTickLabel = [];
        end

        if isBottomRow
            ax.XTickLabelMode = 'auto';
        else
            ax.XTickLabel = [];
        end

        if ~legendAdded
            hCovLegend = plot(ax, NaN, NaN, 'ko', ...
                'MarkerSize',3,'MarkerFaceColor','k');
            hExLegend  = plot(ax, [NaN NaN], [NaN NaN], 'r--', 'LineWidth',1.5);
            hRegLegend = plot(ax, [NaN NaN], [NaN NaN], 'g--', 'LineWidth',1.5);
            lgd = legend(ax, [hCovLegend, hExLegend, hRegLegend], ...
                         {'C_{experimental}','t_{exodus}','t_{regrowth}'}, ...
                         'Location','northwest');
            lgd.Box = 'off';
            legendAdded = true;
        end
    end
end

xlabel(tl, 'Time (hours)');
ylabel(tl, 'Surface coverage (%)');

% ---------- WRITE SUMMARY ----------
Summary = cell2table(summaryRows, ...
    'VariableNames', {'Shear','Conc','ExodusTime_hr','RegrowthTime_hr'});
writetable(Summary, summaryOutFile);
fprintf('\n✅ Exodus/Regrowth summary saved as %s\n', summaryOutFile);

% ---------- WRITE LABELED DATA ----------
T_labeled = cell2table(labeledRows, ...
    'VariableNames', {'Time','Coverage','Shear','Conc','Stage'});
writetable(T_labeled, labeledOutFile);
fprintf('✅ Labeled data saved as %s\n', labeledOutFile);

disp('First few labeled rows:');
head(T_labeled);

% ---------- EXPORT FIGURE ----------
```

```matlab
exportgraphics(fig, 'phase_detection_grid_exo_reg.png', ...
    'Resolution',600);

%% -----------------------------------------------------------------------
%% LOCAL FUNCTION: detectExodusRegrowth_localExtrema
%% -----------------------------------------------------------------------
function [tExo, tReg] = detectExodusRegrowth_localExtrema(t, y)

    t = t(:);
    y = y(:);
    n = numel(t);

    if n < 3
        tExo = NaN;
        tReg = NaN;
        return;
    end

    dropFrac     = 0.20;  % 20% relative drop
    riseFrac     = 0.20;  % 20% relative rise
    maxFutureExo = 5;     % up to 5 future points for exodus
    maxFutureReg = 4;     % up to 4 future points for regrowth

    % --- 1) Exodus detection ---
    tExo = NaN;
    for i = 2:(n-1)
        if y(i) > y(i-1) && y(i) > y(i+1) && y(i) > 0
            remaining = n - i;
            if remaining < 1, continue; end
            futureCount = min(maxFutureExo, remaining);
            futureVals  = y(i+1 : i+futureCount);
            minFuture   = min(futureVals);
            percentDrop = (y(i) - minFuture) / y(i);
            if percentDrop >= dropFrac
                tExo = t(i);
                break;
            end
        end
    end

    % --- 2) Regrowth detection ---
    tReg = NaN;
    if ~isnan(tExo)
        for i = 2:(n-1)
            if t(i) <= tExo
                continue;
            end
            if y(i) < y(i-1) && y(i) < y(i+1) && y(i) > 0
                remaining = n - i;
                if remaining < 1, continue; end
                futureCount = min(maxFutureReg, remaining);
                futureVals  = y(i+1 : i+futureCount);
                maxFuture   = max(futureVals);
                percentRise = (maxFuture - y(i)) / y(i);
                if percentRise >= riseFrac
```

```
                    tReg = t(i);
                    break;
                end
            end
        end
    end
end
```

S1.2. Logistic Growth Model Fitting: This script fits a logistic growth model to the pre-exodus portion of each condition, using exodus times from exodus_regrowth_summary_matlab.csv. It returns estimates of the maximum coverage (C_max), growth rate (k_g), and inflection time (t_mid) for each shear-nutrient condition, and plots data with fitted curves in a tiled grid.

```
%% GROWTH FIT GRID

clear; clc;

set(groot,'DefaultAxesFontName','Times New Roman');
set(groot,'DefaultTextFontName','Times New Roman');

rawDataFile = "sc_v_time_data.csv";
summaryFile = "exodus_regrowth_summary_matlab.csv";

Traw    = readtable(rawDataFile);
Tphases = readtable(summaryFile);

shearVals = unique(Traw.Shear);
concVals  = unique(Traw.Conc);

figure('Name','Growth Fits per Condition', ...
       'Position',[100 100 1200 900], ...
       'Color','w');

tl = tiledlayout(numel(shearVals), numel(concVals), ...
                 'TileSpacing','compact','Padding','compact');

legendAdded = false;
paramRows = {};

for i = 1:numel(shearVals)
    G = shearVals(i);
    for j = 1:numel(concVals)
        N = concVals(j);

        idxP = Tphases.Shear == G & Tphases.Conc == N;
        if ~any(idxP)
            nexttile; axis off; continue;
        end

        t_exo = Tphases.ExodusTime_hr(idxP);
        t_reg = Tphases.RegrowthTime_hr(idxP);

        idx = Traw.Shear == G & Traw.Conc == N;
        tAll = Traw.Time(idx);
        yAll = Traw.Mean(idx);
        if isempty(tAll)
            nexttile; axis off; continue;
        end
        [tAll,ord] = sort(tAll);
        yAll = yAll(ord);

        t_min = min(tAll);  t_max = max(tAll);
```

```
  if ~isnan(t_exo)
      t_min_growth = t_min;
      t_max_growth = t_exo;
  else
      t_min_growth = t_min;
      t_max_growth = t_max;
  end

  segMask = tAll >= t_min_growth & tAll <= t_max_growth;
  t_seg = tAll(segMask);  y_seg = yAll(segMask);

  ax = nexttile;
  hold(ax,'on');

  hData = plot(ax, tAll, yAll, 'ko', ...
      'MarkerFaceColor','k', 'MarkerSize',3, 'LineStyle','none');

  hEx = []; hReg = [];
  if ~isnan(t_exo)
      hEx = xline(ax, t_exo, '--r', 'LineWidth', 1.3);
  end
  if ~isnan(t_reg)
      hReg = xline(ax, t_reg, '--g', 'LineWidth', 1.3);
  end

  if numel(t_seg) >= 4
      growthFun = @(p,t) p(1) ./ (1 + exp(-p(2).*(t - p(3))));
      Cmax0 = max(y_seg); if Cmax0 <= 0, Cmax0 = max(yAll); end
      k0    = 1;
      tmid0 = median(t_seg);
      beta0 = [Cmax0, k0, tmid0];

      opts = statset('nlinfit'); opts.RobustWgtFun = 'bisquare';
      try
          beta_hat = nlinfit(t_seg, y_seg, growthFun, beta0, opts);
          Cmax_hat = beta_hat(1); k_hat = beta_hat(2); tmid_hat = beta_hat(3);

          tFine = linspace(t_min_growth, t_max_growth, 200)';
          yFine = growthFun(beta_hat, tFine);
          hFit  = plot(ax, tFine, yFine, 'b-', 'LineWidth', 2);

          paramRows(end+1,:) = {G,N,t_exo,t_reg,Cmax_hat,k_hat,tmid_hat};
      catch
          hFit = plot(ax, NaN, NaN);
      end
  else
      hFit = plot(ax, NaN, NaN);
  end

  xlim(ax, [0 10]);
  xticks([0 2 4 6 8 10]);
  ylim(ax, [0 100]);
  yticks([0 20 40 60 80 100]);
box(ax,'on');
```

```
ax.XGrid = 'off';
ax.YGrid = 'off';

isLeftColumn = (j == 1);
isBottomRow  = (i == numel(shearVals));

if isLeftColumn
    ax.YTickLabelMode = 'auto';
else
    ax.YTickLabel = [];
end

if isBottomRow
    ax.XTickLabelMode = 'auto';
else
    ax.XTickLabel = [];
end

        title(ax, sprintf('\\gamma = %g s^{-1}, TSB = %.2fx', G, N), FontSize=10);
        if ~legendAdded
            hCov  = plot(ax, NaN, NaN, 'ko', 'MarkerFaceColor','k', 'MarkerSize', 2);
            hFitL = plot(ax, NaN, NaN, 'b-', 'LineWidth',2);
            hExL  = plot(ax, NaN, NaN, 'r--', 'LineWidth',1.3);
            hRegL = plot(ax, NaN, NaN, 'g--', 'LineWidth',1.3);

            lgd = legend(ax, [hCov,hFitL,hExL,hRegL], ...
                {'C_{experimental}','C_{growth}','t_{exodus}','t_{regrowth}'}, ...
                'Location','northwest');
            lgd.Box = 'off';
            legendAdded = true;
        end
    end
end
xlabel(tl, 'Time (hours)','fontsize',12);
ylabel(tl, 'Surface coverage (%)','fontsize',12);
exportgraphics(gcf, "growth_fits_grid.png", 'Resolution', 600);

% ---- Save growth parameter table ----
GrowthParamTable = cell2table(paramRows, ...
    'VariableNames', {'Shear','Conc','t_exo','t_reg','C_max','k_g','t_mid'});
writetable(GrowthParamTable, "growth_param_table.csv");
fprintf('Saved growth_param_table.csv\n');
```

S1.3. Exodus Model Fitting: This script fits an exponential decay model to the exodus segment identified for each condition, starting at the detected exodus time and ending at either the regrowth onset or final timepoint. It estimates C_exo, C_inf, and the exodus rate constant k_d, and computes R^2 and RMSE diagnostics.

```
%% EXODUS FIT GRID
%  Model:
%     C(t) = C_inf + (C_exo - C_inf) * exp( -k_d * (t - t_exo) )

clear; clc;

set(groot,'DefaultAxesFontName','Times New Roman');
set(groot,'DefaultTextFontName','Times New Roman');

rawDataFile  = "sc_v_time_data.csv";
summaryFile  = "exodus_regrowth_summary_matlab.csv";

Traw    = readtable(rawDataFile);
Tphases = readtable(summaryFile);

shearVals = unique(Traw.Shear);
concVals  = unique(Traw.Conc);

fig = figure('Name','Exodus Fits per Condition', ...
             'Position',[100 100 1200 900], ...
             'Color','w');

tl = tiledlayout(numel(shearVals), numel(concVals), ...
                 'TileSpacing','compact','Padding','compact');

legendAdded = false;
paramRows = {};

for i = 1:numel(shearVals)
    G = shearVals(i);
    for j = 1:numel(concVals)
        N = concVals(j);

        % --- Phase times for this condition ---
        rowMask = (Tphases.Shear == G) & (Tphases.Conc == N);
        if ~any(rowMask)
            ax = nexttile; axis(ax,'off');
            continue;
        end

        t_exo = Tphases.ExodusTime_hr(rowMask);
        t_reg = Tphases.RegrowthTime_hr(rowMask);

        % --- Raw data for this condition ---
        idx  = (Traw.Shear == G) & (Traw.Conc == N);
        tAll = Traw.Time(idx);
        yAll = Traw.Mean(idx);

        [tAll, ord] = sort(tAll);
```

```
yAll = yAll(ord);

ax = nexttile;
hold(ax,'on');

% Experimental data
hData = plot(ax, tAll, yAll, 'ko', ...
    'MarkerFaceColor','k', ...
    'MarkerSize',3, ...
    'LineStyle','none');
xlim(ax,[0 10]);
ylim(ax,[0 100]);
xticks(ax,[0 2 4 6 8 10]);
yticks(ax,[0 20 40 60 80 100]);
box(ax,'on');
ax.XGrid = 'off';
ax.YGrid = 'off';

if j == 1
    ax.YTickLabelMode = 'auto';
else
    ax.YTickLabel = [];
end

if i == numel(shearVals)
    ax.XTickLabelMode = 'auto';
else
    ax.XTickLabel = [];
end

title(ax, sprintf('\\gamma = %g s^{-1}, TSB = %.2fx', G, N), FontSize=10);

% --- If NO exodus: annotate and store NaN
if isnan(t_exo)
    text(ax, 5, 50, 'No Exodus', 'Color','r', ...
         'HorizontalAlignment','center');
    paramRows(end+1,:) = {G, N, NaN, t_reg, NaN, NaN, NaN, 0, NaN, NaN};

    continue;
end

% --- Define exodus segment [t_exo, t_reg] or [t_exo, t_max] ---
if ~isnan(t_reg) && (t_reg > t_exo)
    t_min_exo = t_exo;
    t_max_exo = t_reg;
else
    t_min_exo = t_exo;
    t_max_exo = max(tAll);
end

segMask = (tAll >= t_min_exo) & (tAll <= t_max_exo);
t_seg = tAll(segMask);
y_seg = yAll(segMask);

% Mark Exodus & Regrowth times
```

```
        hEx  = xline(ax, t_exo, 'r--', 'LineWidth',2.2);
        hReg = [];
        if ~isnan(t_reg)
            hReg = xline(ax, t_reg, 'g--', 'LineWidth',2.2);
        end


        if numel(t_seg) < 3
            paramRows(end+1,:) = {G, N, t_exo, t_reg, NaN, NaN, NaN, numel(t_seg),
NaN, NaN};
            continue;
        end
        [~, idxNear] = min(abs(tAll - t_exo));
        C_exo = yAll(idxNear);

        % Exodus model:
        %   C(t) = C_inf + (C_exo - C_inf) * exp( -k_d * (t - t_exo) )
        exoFun = @(p,t) p(1) + (C_exo - p(1)) .* exp(-p(2) .* (t - t_exo));
        C_inf0 = min(y_seg);
        kd0    = 0.5;
        beta0  = [C_inf0, kd0];

        opts = statset('nlinfit');
        opts.RobustWgtFun = 'bisquare';

        try
            beta_hat   = nlinfit(t_seg, y_seg, exoFun, beta0, opts);
            C_inf_hat  = beta_hat(1);
            k_d_hat    = beta_hat(2);

            % Smooth fitted exodus curve
            tFine = linspace(t_min_exo, t_max_exo, 200)';
            yFine = exoFun(beta_hat, tFine);

            hFit = plot(ax, tFine, yFine, 'r-', 'LineWidth',2);

            % Statistical Metrics
            residuals = y_seg - exoFun(beta_hat, t_seg);
            SS_res    = sum(residuals.^2);
            SS_tot    = sum((y_seg - mean(y_seg)).^2);
            R2        = 1 - SS_res/SS_tot;
            RMSE      = sqrt(mean(residuals.^2));

            paramRows(end+1,:) = {G, N, t_exo, t_reg, C_exo, C_inf_hat, ...
                                  k_d_hat, numel(t_seg), R2, RMSE};
        catch
            hFit = plot(ax, NaN,NaN);
            paramRows(end+1,:) = {G, N, t_exo, t_reg, C_exo, NaN, NaN, ...
                                  numel(t_seg), NaN, NaN};
        end

        if ~legendAdded
            hC      = plot(ax, NaN,NaN,'ko','MarkerFaceColor','k','MarkerSize',3);
            hFitL   = plot(ax, NaN,NaN,'r-','LineWidth',2);
            hExL    = plot(ax, NaN,NaN,'r--','LineWidth',1.5);
```

```
            hRegL  = plot(ax, NaN,NaN,'g--','LineWidth',1.5);
            lgd = legend(ax, [hC,hFitL,hExL,hRegL], ...
                {'C_{experimental}','C_{exodus}','t_{exodus}','t_{regrowth}'}, ...
                'Location','northwest');
            lgd.Box = 'off';
            legendAdded = true;
        end
    end
end
xlabel(tl,'Time (hours)','fontsize',12);
ylabel(tl,'Surface coverage (%)','fontsize',12);
exportgraphics(fig,'exodus_fits_grid.png','Resolution',600);

%% ---- Save parameter table ----
ExoParamTable = cell2table(paramRows, ...
    'VariableNames', {'Shear','Conc','t_exo','t_reg', ...
                      'C_exo','C_inf','k_d','nPoints','R2','RMSE'});
writetable(ExoParamTable,"exodus_param_table.csv");
fprintf('Saved exodus_param_table.csv\n');
```

S1.4. Regrowth Model Fitting: This script fits an exponential recovery model to the regrowth segment for each condition, beginning at the detected regrowth onset and extending to the final available timepoints. It estimates the regrowth asymptote y_inf, the regrowth starting value y0, and the regrowth rate constant k. The script generates individual regrowth-fit panels for all shear-nutrient combinations and records fitted parameters for downstream lifecycle assembly.

```
%% REGROWTH FIT GRID
%  Model:
%     y(t) = y_inf - (y_inf - y0) * exp( -k * t_rel )

clear; clc;

set(groot,'DefaultAxesFontName','Times New Roman');
set(groot,'DefaultTextFontName','Times New Roman');

rawFile     = "sc_v_time_data.csv";
summaryFile = "exodus_regrowth_summary_matlab.csv";
labelFile   = "labeled_biofilm_data_matlab.csv";

Traw     = readtable(rawFile);
Tsummary = readtable(summaryFile);
Tlabeled = readtable(labelFile);

shearVals = unique(Traw.Shear);
concVals  = unique(Traw.Conc);

fig = figure('Name','Regrowth Fits per Condition', ...
             'Position',[100 100 1300 900], ...
             'Color','w');

tl = tiledlayout(numel(shearVals), numel(concVals), ...
                 'TileSpacing','compact','Padding','compact');

legendAdded = false;
paramRows = {};

for i = 1:numel(shearVals)
    G = shearVals(i);
    for j = 1:numel(concVals)
        N = concVals(j);

        ax = nexttile; hold(ax,'on');

        %% ---- extract phase times ----
        idxSum = (Tsummary.Shear == G) & (Tsummary.Conc == N);
        if ~any(idxSum)
            axis(ax,'off');
            continue;
        end

        t_exo = Tsummary.ExodusTime_hr(idxSum);
        t_reg = Tsummary.RegrowthTime_hr(idxSum);
```

```matlab
%% ---- raw data ----
idx = (Traw.Shear == G) & (Traw.Conc == N);
tAll = Traw.Time(idx);
yAll = Traw.Mean(idx);
[tAll, ord] = sort(tAll);
yAll = yAll(ord);

plot(ax, tAll, yAll, 'ko', ...
     'MarkerFaceColor','k', 'MarkerSize',3, 'LineStyle','none');

%% ---- vertical lines ----
if ~isnan(t_exo)
    xline(ax, t_exo, 'r--', 'LineWidth',2.2);
end
if ~isnan(t_reg)
    xline(ax, t_reg, 'g--', 'LineWidth',2.2);
end

%% ---- no regrowth
if isnan(t_reg)
    text(ax, 5, 50, 'No Regrowth', 'Color','r', ...
        'HorizontalAlignment','center');
end

%% ---- extract regrowth points ----
isReg = (Tlabeled.Shear == G) & (Tlabeled.Conc == N) & ...
        (categorical(Tlabeled.Stage) == "Regrowth");

tR = Tlabeled.Time(isReg);
yR = Tlabeled.Coverage(isReg);
[tR, ordR] = sort(tR);
yR = yR(ordR);

doFit = (~isnan(t_reg) && numel(tR) >= 3);

if doFit
    t0 = tR(1);
    t_rel = tR - t0;
    y0 = yR(1);

    y_inf0 = max(yR);
    if y_inf0 < y0
        y_inf0 = y0 + 5;
    end
    k0 = 1/max(t_rel+eps);

    modelFun = @(p,t) p(1) - (p(1)-p(2)) .* exp(-p(3).*t);
    costFun = @(p) regrowth_cost(p, t_rel, yR, modelFun);

    opts = optimset('Display','off');
    p_hat = fminsearch(costFun, [y_inf0, y0, k0], opts);

    y_inf_hat = p_hat(1);
    y0_hat    = p_hat(2);
    k_hat     = p_hat(3);
```

```matlab
            % smooth regrowth curve
            t_rel_fine = linspace(0, max(tAll)-t0, 200)';
            y_fit = modelFun(p_hat, t_rel_fine);
            t_abs = t0 + t_rel_fine;

            plot(ax, t_abs, y_fit, 'g-', 'LineWidth',2);

            % store parameters
            paramRows(end+1,:) = {G,N,t_exo,t_reg,t0,y_inf_hat,y0_hat,k_hat};

        else

            paramRows(end+1,:) = {G,N,t_exo,t_reg,NaN,NaN,NaN,NaN};
        end

        xlim(ax,[0 10]);
        ylim(ax,[0 100]);
        xticks(ax,[0 2 4 6 8 10]);
        yticks(ax,[0 20 40 60 80 100]);
        box(ax,'on');

        if j == 1
            ax.YTickLabelMode = "auto";
        else
            ax.YTickLabel = [];
        end

        if i == numel(shearVals)
            ax.XTickLabelMode = "auto";
        else
            ax.XTickLabel = [];
        end

        title(ax, sprintf('\\gamma = %g s^{-1}, TSB = %.2fx', G, N), FontSize= 10);

        if ~legendAdded && doFit
            hC     = plot(ax,NaN,NaN,'ko','MarkerFaceColor','k','MarkerSize',2);
            hFitL  = plot(ax,NaN,NaN,'g-','LineWidth',2);
            hExL   = plot(ax,NaN,NaN,'r--','LineWidth',1.5);
            hRegL  = plot(ax,NaN,NaN,'g--','LineWidth',1.5);

            lgd = legend(ax,[hC,hFitL,hExL,hRegL], ...
                {'C_{experimental}','C_{regrowth}','t_{exodus}','t_{regrowth}'}, ...
                'Location','northwest');
            lgd.Box = "off";
            legendAdded = true;
        end

    end
end

xlabel(tl,'Time (hours)','fontsize',12);
ylabel(tl,'Surface coverage (%)','fontsize',12);
```

```matlab
exportgraphics(fig,'regrowth_fits_grid.png','Resolution',600);


%% ---- local function ----
function J = regrowth_cost(p, t_rel, y_obs, fn)
    y_inf = p(1);
    y0    = p(2);
    k     = p(3);

    y_pred = fn(p,t_rel);
    SSE = sum((y_obs - y_pred).^2);

    penalty = 0;
    if y_inf < y0
        penalty = penalty + 1e4*(y0-y_inf)^2;
    end
    if k < 0
        penalty = penalty + 1e4*k^2;
    end

    J = SSE + penalty;
end

% ---- Save regrowth parameter table ----
RegrowthParamTable = cell2table(paramRows, ...
    'VariableNames', {'Shear','Conc','t_exo','t_reg','t0_reg','y_inf','y0','k'});
writetable(RegrowthParamTable, "regrowth_param_label_based.csv");
fprintf('Saved regrowth_param_label_based.csv\n');
```

S1.5. Lifecycle Parameter Table and Interpolants: This script compiles the detected phase times and phase-specific kinetic parameters into a single lifecycle parameter table, then constructs scatteredInterpolant objects for each parameter across the shear-nutrient space. These interpolants are used to predict lifecycle dynamics at any point within (or near) the experimental grid.

```
%% BUILD LIFECYCLE PARAMETER TABLE

clear; clc;

summaryFile  = "exodus_regrowth_summary_matlab.csv";
growthFile   = "growth_param_table.csv";
exodusFile   = "exodus_param_table.csv";
regrowthFile = "regrowth_param_label_based.csv";

% READ INPUT TABLES
Tsum  = readtable(summaryFile);   % Shear, Conc, ExodusTime_hr, RegrowthTime_hr
Tg    = readtable(growthFile);    % Shear, Conc, C_max, k_g, t_mid, ...
Te    = readtable(exodusFile);    % Shear, Conc, C_exo, C_inf, k_d, ...
Tr    = readtable(regrowthFile);  % Shear, Conc, ExodusTime_hr, RegrowthTime_hr,
t0_reg, y_inf, y0, k, ...

reqSum = {'Shear','Conc','ExodusTime_hr','RegrowthTime_hr'};
assert(all(ismember(reqSum, Tsum.Properties.VariableNames)), ...
    'Summary file missing one or more required columns.');

reqG = {'Shear','Conc','C_max','k_g','t_mid'};
assert(all(ismember(reqG, Tg.Properties.VariableNames)), ...
    'Growth param file missing one or more required columns.');

reqE = {'Shear','Conc','C_exo','C_inf','k_d'};
assert(all(ismember(reqE, Te.Properties.VariableNames)), ...
    'Exodus param file missing one or more required columns.');

reqR = {'Shear','Conc','t0_reg','y_inf','y0','k'};
assert(all(ismember(reqR, Tr.Properties.VariableNames)), ...
    'Regrowth param file missing one or more required columns.');
Tsum_keep = Tsum(:, {'Shear','Conc','ExodusTime_hr','RegrowthTime_hr'});
Tg_keep   = Tg(:,   {'Shear','Conc','C_max','k_g','t_mid'});
Te_keep   = Te(:,   {'Shear','Conc','C_exo','C_inf','k_d'});
Tr_keep   = Tr(:,   {'Shear','Conc','t0_reg','y_inf','y0','k'});

%JOIN ALL TABLES
T1 = innerjoin(Tsum_keep, Tg_keep, 'Keys', {'Shear','Conc'});
T2 = innerjoin(T1,        Te_keep, 'Keys', {'Shear','Conc'});
LifecycleParams = innerjoin(T2,    Tr_keep, 'Keys', {'Shear','Conc'});

disp('=== MASTER LIFECYCLE PARAMETER TABLE ===');
disp(LifecycleParams);
writetable(LifecycleParams, "lifecycle_param_table.csv");
fprintf('Saved lifecycle_param_table.csv');

% BUILD INTERPOLANTS FOR EACH PARAMETER OVER (Shear, Conc)
```

```
LifecycleParams = readtable("lifecycle_param_table.csv");

Gv = LifecycleParams.Shear;
Nv = LifecycleParams.Conc;

method = 'nearest';
extrap = 'nearest';
F = struct();

% Phase times
F.t_exo  = scatteredInterpolant(Gv, Nv, LifecycleParams.ExodusTime_hr,   method,
extrap);
F.t_reg  = scatteredInterpolant(Gv, Nv, LifecycleParams.RegrowthTime_hr, method,
extrap);

% Growth parameters
F.C_max  = scatteredInterpolant(Gv, Nv, LifecycleParams.C_max,           method,
extrap);
F.k_g    = scatteredInterpolant(Gv, Nv, LifecycleParams.k_g,             method,
extrap);
F.t_mid  = scatteredInterpolant(Gv, Nv, LifecycleParams.t_mid,           method,
extrap);

% Exodus parameters
F.C_exo  = scatteredInterpolant(Gv, Nv, LifecycleParams.C_exo,           method,
extrap);
F.C_inf  = scatteredInterpolant(Gv, Nv, LifecycleParams.C_inf,           method,
extrap);
F.k_d    = scatteredInterpolant(Gv, Nv, LifecycleParams.k_d,             method,
extrap);

% Regrowth parameters
F.t0_reg = scatteredInterpolant(Gv, Nv, LifecycleParams.t0_reg,          method,
extrap);
F.y_inf  = scatteredInterpolant(Gv, Nv, LifecycleParams.y_inf,           method,
extrap);
F.y0_reg = scatteredInterpolant(Gv, Nv, LifecycleParams.y0,              method,
extrap);
F.kR     = scatteredInterpolant(Gv, Nv, LifecycleParams.k,               method,
extrap);

save("lifecycle_interpolants.mat","F");
fprintf('Saved lifecycle_interpolants.mat ');
```

S1.6. Lifecycle Model Visualization and Prediction

S1.6.1. Lifecycle vs. Experimental Data: This script reconstructs the full lifecycle (growth-exodus-regrowth) for each measured shear-nutrient condition using the interpolated parameter surfaces, and overlays the model prediction on experimental data in a tiled grid, with segments color-coded by phase.

```
% Lifecycle model vs data for all original (Shear, Conc) conditions
% Growth = blue, Exodus = red, Regrowth = green

clear; clc;

%% ---------- CONFIG ----------
rawFile    = "sc_v_time_data.csv";          % experimental data
paramFile  = "lifecycle_param_table.csv";   % list of (Shear, Conc)
interpFile = "lifecycle_interpolants.mat";  % struct F with interpolants

tFine = (0:0.05:10)';

set(groot,'DefaultAxesFontName','Times New Roman');
set(groot,'DefaultTextFontName','Times New Roman');

%% ---------- LOAD DATA & INTERPOLANTS ----------
Traw            = readtable(rawFile);
LifecycleParams = readtable(paramFile);
load(interpFile, "F");

shearVals = unique(LifecycleParams.Shear);
concVals  = unique(LifecycleParams.Conc);

%% ---------- PLOTS: ALL ORIGINAL CONDITIONS (SEGMENT-COLORED) ----------
fig = figure('Name','Lifecycle model vs data (all original conditions)', ...
             'Position',[100 100 1300 900], 'Color','w');

tl = tiledlayout(numel(shearVals), numel(concVals), ...
                 "TileSpacing","compact", "Padding","compact");

legendAdded = false;

for i = 1:numel(shearVals)
    G = shearVals(i);
    for j = 1:numel(concVals)
        N = concVals(j);

        % --- Experimental data for this (G, N) ---
        idxCond = (Traw.Shear == G) & (Traw.Conc == N);
        t_exp   = Traw.Time(idxCond);
        y_exp   = Traw.Mean(idxCond);

        ax = nexttile;
        hold(ax,'on');

        title(ax, sprintf('\\gamma = %g s^{-1}, TSB = %.2fx', G, N), ...
              'FontSize',10);
```

```
xlim(ax,[0 10]);
ylim(ax,[0 100]);
xticks(ax,0:2:10);
yticks(ax,0:20:100);
box(ax,'on');
ax.XGrid = 'off';
ax.YGrid = 'off';


if j == 1
    ax.YTickLabelMode = 'auto';
else
    ax.YTickLabel = [];
end

if i == numel(shearVals)
    ax.XTickLabelMode = 'auto';
else
    ax.XTickLabel = [];
end

if ~isempty(t_exp)
    [t_exp, ord] = sort(t_exp);
    y_exp = y_exp(ord);
    hData = plot(ax, t_exp, y_exp, 'ko', ...
                 'MarkerFaceColor','k', ...
                 'MarkerSize',3, ...
                 'LineStyle','none');
else
    hData = [];
end


t_exo  = F.t_exo(G, N);
t0_reg = F.t0_reg(G, N);
y_model = predict_lifecycle_global(tFine, G, N, F);

if all(isnan(y_model))
    if isempty(t_exp)
        text(ax,5,80,'No data / model','Color','r', ...
             'HorizontalAlignment','center');
    end
    continue;
end

% Growth: t <= t_exo   or no exodus
if isnan(t_exo)
    growthMask = true(size(tFine));
else
    growthMask = tFine <= t_exo;
end

% Exodus: t_exo < t <= t0_reg
if isnan(t_exo)
```

```
        exoMask = false(size(tFine));
    elseif isnan(t0_reg)
        exoMask = tFine > t_exo;
    else
        exoMask = (tFine > t_exo) & (tFine <= t0_reg);
    end

    % Regrowth: t > t0_reg (if exists)
    if isnan(t0_reg)
        regMask = false(size(tFine));
    else
        regMask = tFine > t0_reg;
    end


    hGrow = []; hExo = []; hReg = [];

    if any(growthMask)
        hGrow = plot(ax, tFine(growthMask), y_model(growthMask), ...
                     'b-', 'LineWidth', 2);
    end
    if any(exoMask)
        hExo = plot(ax, tFine(exoMask), y_model(exoMask), ...
                    'r-', 'LineWidth', 2);
    end
    if any(regMask)
        hReg = plot(ax, tFine(regMask), y_model(regMask), ...
                    'g-', 'LineWidth', 2);
    end

    if ~isnan(t_exo)
        xline(ax, t_exo, 'r--', 'LineWidth', 1.2);
    end
    if ~isnan(t0_reg)
        xline(ax, t0_reg, 'g--', 'LineWidth', 1.2);
    end

    if ~legendAdded

        if isempty(hData)
            hData = plot(ax, NaN,NaN, 'ko', 'MarkerFaceColor','k',
'MarkerSize',3);
        end
        if isempty(hGrow)
            hGrow = plot(ax, NaN,NaN, 'b-', 'LineWidth',2);
        end
        if isempty(hExo)
            hExo = plot(ax, NaN,NaN, 'r-', 'LineWidth',2);
        end
        if isempty(hReg)
            hReg = plot(ax, NaN,NaN, 'g-', 'LineWidth',2);
        end

        lgd = legend(ax, [hData, hGrow, hExo, hReg], ...
                     {'C_{Experimental}','C_{Growth}', ...
```

```
                               'C_{Exodus}','C_{Regrowth}'}, ...
                              'Location','northwest');
            lgd.Box = 'off';
            legendAdded = true;
        end

    end
end

xlabel(tl, 'Time (hours)', 'FontSize', 12);
ylabel(tl, 'Surface coverage (%)', 'FontSize', 12);

exportgraphics(fig, "lifecycle_all_conditions_colored.png", ...
               "Resolution", 600);

fprintf('Plotted color-segmented lifecycle model vs data for all original
conditions.');


%% LIFECYCLE PREDICTOR (USES INTERPOLATED PARAMETERS)
function y = predict_lifecycle_global(t_vec, G, N, F)
    t_vec = t_vec(:);
    y     = NaN(size(t_vec));


    t_exo   = F.t_exo(G, N);
    t_reg   = F.t_reg(G, N);
    C_max   = F.C_max(G, N);
    k_g     = F.k_g(G, N);
    t_mid   = F.t_mid(G, N);

    C_exo   = F.C_exo(G, N);
    C_inf   = F.C_inf(G, N);
    k_d     = F.k_d(G, N);

    t0_reg  = F.t0_reg(G, N);
    y_inf   = F.y_inf(G, N);
    y0_reg  = F.y0_reg(G, N);
    kR      = F.kR(G, N);

    if any(isnan([C_max, k_g, t_mid]))
        return;
    end

    growthFun = @(t) C_max ./ (1 + exp(-k_g .* (t - t_mid)));

    hasExo = ~(isnan(t_exo) || isnan(C_inf) || isnan(k_d));
    if hasExo
        if isnan(C_exo)
            C_exo = growthFun(t_exo);
        end
        exoFun = @(t) C_inf + (C_exo - C_inf) .* exp(-k_d .* max(0, t - t_exo));
    end

    hasReg = ~(isnan(t0_reg) || isnan(y_inf) || isnan(y0_reg) || isnan(kR));
```

```
    if hasReg
        regFun = @(t) y_inf - (y_inf - y0_reg) .* exp(-kR .* max(0, t - t0_reg));
    end

    for k = 1:numel(t_vec)
        tk = t_vec(k);

        if ~hasExo || tk <= t_exo
            y(k) = growthFun(tk);
        elseif ~hasReg || tk <= t0_reg
            y(k) = exoFun(tk);
        else
            y(k) = regFun(tk);
        end
    end
end
```

S1.6.2. Interactive Lifecycle Prediction at User-Defined Conditions: This script allows the user to input shear rate and TSB concentration within the experimental range and generates a predicted lifecycle curve using the interpolated parameters, with phase segments and predicted exodus/regrowth times indicated.

```
%% INTERPOLATED LIFECYCLE PLOT BASED ON INPUT SHEAR & CONCENTRATION
% Growth = blue
% Exodus = red
% Regrowth = green
% Also plots predicted t_exodus and t_regrowth

clear; clc;

set(groot,'DefaultAxesFontName','Times New Roman');
set(groot,'DefaultTextFontName','Times New Roman');

paramFile   = "lifecycle_param_table.csv";
interpFile  = "lifecycle_interpolants.mat";

LifecycleParams = readtable(paramFile);
load(interpFile, "F");

shearVals = unique(LifecycleParams.Shear);
concVals  = unique(LifecycleParams.Conc);

Gmin = min(shearVals); Gmax = max(shearVals);
Nmin = min(concVals);  Nmax = max(concVals);

fprintf('     BIOFILM LIFECYCLE PREDICTION');

%% ---------- USER INPUT ----------
G_interp = input(sprintf(' Enter shear rate γ (%.1f–%.1f): ', Gmin, Gmax));
N_interp = input(sprintf(' Enter concentration (%.2f–%.2f): ', Nmin, Nmax));

if G_interp < Gmin || G_interp > Gmax
    warning('Shear is outside experimental range — model will extrapolate.');
end
if N_interp < Nmin || N_interp > Nmax
    warning('Concentration is outside experimental range — model will extrapolate.');
end

fprintf('Using G = %.3f s^-1, N = %.3f xTSB', G_interp, N_interp);

%% ---------- PREPARE TIME VECTOR ----------
tFine = (0:0.05:10)';

%% ---------- GET PARAMETERS FOR PHASE BOUNDARIES ----------
t_exo  = F.t_exo(G_interp, N_interp);
t0_reg = F.t0_reg(G_interp, N_interp);

%% ---------- COMPUTE MODEL ----------
y_pred = predict_lifecycle_global(tFine, G_interp, N_interp, F);

if isnan(t_exo)
```

```
    growthMask = true(size(tFine));
    exoMask    = false(size(tFine));
    regMask    = false(size(tFine));
else
    growthMask = tFine <= t_exo;

    if isnan(t0_reg)
        exoMask = tFine > t_exo;
        regMask = false(size(tFine));
    else
        exoMask = (tFine > t_exo) & (tFine <= t0_reg);
        regMask = tFine > t0_reg;
    end
end

figure('Name','Interpolated lifecycle', 'Position',[200 200 650 500], 'Color','w');
hold on; box on;

% Blue = Growth
if any(growthMask)
    plot(tFine(growthMask), y_pred(growthMask), 'b-', 'LineWidth', 3);
end

% Red = Exodus
if any(exoMask)
    plot(tFine(exoMask), y_pred(exoMask), 'r-', 'LineWidth', 3);
end

% Green = Regrowth
if any(regMask)
    plot(tFine(regMask), y_pred(regMask), 'g-', 'LineWidth', 3);
end

% Vertical lines
if ~isnan(t_exo)
    xline(t_exo, 'r--', 'LineWidth', 2);
end
if ~isnan(t0_reg)
    xline(t0_reg, 'g--', 'LineWidth', 2);
end

xlabel('Time (hours)');
ylabel('Surface coverage (%)');
title(sprintf('Predicted Lifecycle (γ = %.1f s^{-1}, TSB = %.2fx)', ...
              G_interp, N_interp));

xlim([0 10]);
ylim([0 100]);
set(gca,'FontSize',12);
hGrow = plot(NaN,NaN,'b-','LineWidth',3);
hExo  = plot(NaN,NaN,'r-','LineWidth',3);
hReg  = plot(NaN,NaN,'g-','LineWidth',3);
hExVL = plot(NaN,NaN,'r--','LineWidth',2);
hRgVL = plot(NaN,NaN,'g--','LineWidth',2);
```

```
legend([hGrow hExo hReg hExVL hRgVL], ...
    {'C_{growth}','C_{exodus}','C_{regrowth}','t_{exodus}','t_{regrowth}'}, ...
    'Location','northwest','Box','off');

fprintf('Predicted t_exodus = %.3f hours\n', t_exo);
fprintf('Predicted t_regrowth = %.3f hours\n', t0_reg);
fprintf('\n✅ Plot complete.\n');

function y = predict_lifecycle_global(t_vec, G, N, F)

    t_vec = t_vec(:);
    y     = NaN(size(t_vec));

    t_exo   = F.t_exo(G, N);
    t0_reg  = F.t0_reg(G, N);
    C_max   = F.C_max(G, N);
    k_g     = F.k_g(G, N);
    t_mid   = F.t_mid(G, N);

    C_exo   = F.C_exo(G, N);
    C_inf   = F.C_inf(G, N);
    k_d     = F.k_d(G, N);

    y_inf   = F.y_inf(G, N);
    y0_reg  = F.y0_reg(G, N);
    kR      = F.kR(G, N);

    if any(isnan([C_max, k_g, t_mid]))
        return;
    end

    growthFun = @(t) C_max ./ (1 + exp(-k_g .* (t - t_mid)));

    hasExo = ~(isnan(t_exo) || isnan(C_inf) || isnan(k_d));
    if hasExo
        if isnan(C_exo)
            C_exo = growthFun(t_exo);
        end
        exoFun = @(t) C_inf + (C_exo - C_inf) .* exp(-k_d .* max(0, t - t_exo));
    end

    hasReg = ~(isnan(t0_reg) || isnan(y_inf) || isnan(y0_reg) || isnan(kR));
    if hasReg
        regFun = @(t) y_inf - (y_inf - y0_reg) .* exp(-kR .* max(0, t - t0_reg));
    end

    for k = 1:numel(t_vec)
        tk = t_vec(k);

        if ~hasExo || tk <= t_exo
            y(k) = growthFun(tk);

        elseif ~hasReg || tk <= t0_reg
            y(k) = exoFun(tk);
```

```
        else
            y(k) = regFun(tk);
        end
    end
end
```

S1.7. MATLAB Version: All scripts were developed and tested in **MATLAB R2024a** with standard toolboxes. No external libraries are required.
